# Structure–Activity Relationships of Anabaenopeptins as Carboxypeptidase and Phosphatase Inhibitors

**DOI:** 10.1021/acschembio.5c00791

**Published:** 2026-01-21

**Authors:** Megan L. Quandt, Judy Westrick, Jeremy J. Kodanko

**Affiliations:** Department of Chemistry, 2954 Wayne State University, 5101 Cass Avenue, Detroit, Michigan 48202, United States

## Abstract

Anabaenopeptins are a family of cyanobacterial cyclic peptides that display potent enzyme inhibition, particularly against carboxypeptidases A and B, as well as the serine/threonine phosphatases PP1 and PP2A. Defined by a 19-membered macrocyclic ring and a ureido-linked exocyclic amino acid, these compounds vary considerably in their amino acid composition, influencing both potency and selectivity. This review compiles published IC_50_ values for natural and synthetic anabaenopeptins, organizing them by enzyme target, and highlighting recurring structural motifs that drive inhibitory activity. Through this comparative analysis, we identify emerging trends in structure–activity relationships and underscore gaps in assay standardization and structural validation. These insights provide a critical foundation for advancing the biological evaluation of anabaenopeptins as environmental contaminants, mechanistic probes, and candidate scaffolds for therapeutic development.

## Graphical abstract

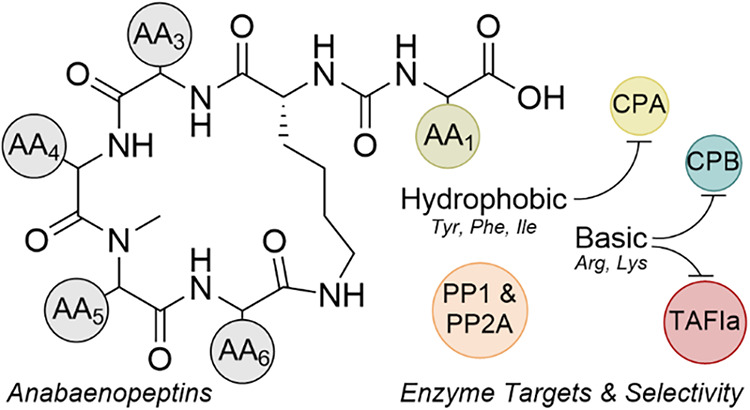

## 1 Introduction

Anabaenopeptins (ABPs) are cyclic peptides produced by cyanobacteria that frequently occur in freshwater ecosystems worldwide, often alongside more recognized cyanotoxins such as microcystins.^1–4^ Their structural diversity and ability to potently inhibit enzymes *in vitro* have made them a growing focus of chemical biology research, with potential applications as mechanistic probes and scaffolds for the design of inhibitors.^5–7^ At the same time, the widespread occurrence of ABPs during harmful algal blooms raises the possibility that environmental exposure could have meaningful effects on biological processes.^8^ Most current knowledge of ABP activity comes from IC_50_ values measured in biochemical assays, yet the physiological implications of these interactions, whether in digestion, clot regulation, or cellular signaling, remain unexplored.^9^ Bridging this gap requires consolidating and critically evaluating existing enzyme inhibition data to assess not only structure–activity relationships (SARs) but also the extent to which *in vitro* potency may translate to effects in organisms.

ABPs feature a conserved 19-membered macrocyclic ring and a ureido-linked exocyclic amino acid.^10^ This scaffold supports remarkable diversity, including proteinogenic and nonproteinogenic residues with over 120 congeners identified across different cyanobacterial genera, with substitutions at nearly every ring position.^11^ These seemingly subtle changes strongly influence binding to targets such as carboxypeptidases and protein phosphatases, making ABPs an ideal platform for probing enzyme recognition and inhibitor design.^12,13^ What remains uncertain is whether the same structural features that drive potency *in vitro* carry biological relevance in humans or other organisms exposed to these compounds.

Despite the rising number of reported ABPs, comparisons of IC_50_ values remain challenging. Many studies do not include benchmark inhibitors, making it harder to compare data across studies from different laboratories. Structural assignments based only on MS/MS often leave stereochemistry uncertain with isomeric residues like Ile, *allo*-Ile, and Leu.^14–16^ Quantification methods introduce additional variability: concentrations are sometimes estimated indirectly or reported in μg/mL without adjusting for molecular weight, which can influence interpretation of potency data.^17^ The design of assays also varies widely in enzyme source, substrate choice, buffer composition, and detection methods, which makes comparisons across different studies difficult.^12,18^ These uncertainties highlight a key issue: we know that ABPs inhibit many enzymes but do not fully understand the implications of this activity on human health or its effects in the environment.

This account compiles IC_50_ values for ABPs and related cyclic peptides, categorized by their respective enzyme targets. The most extensive data are available for metallocarboxypeptidases: carboxypeptidases A (CPA), B (CPB), and the activated thrombin-activatable fibrinolysis inhibitor (TAFIa).^9,12,17^ Inhibition of serine/threonine phosphatases PP1 and PP2A has been studied less thoroughly and is included mainly for comparison.^13,17,19^ This review does not aim to be a comprehensive survey of all cyanobacterial metabolites. Instead, it focuses on ABPs and closely related peptides, highlighting enzyme inhibition profiles, SARs, and current limitations in applying these findings to human health. Given the scope and uneven availability of activity data, this review primarily focuses on consolidating current knowledge of biological activity and, where possible, defining SARs. By consolidating these data sets, we aim to identify common structural patterns, highlight unusual outliers, and evaluate the observed potencies in biochemical terms and potential environmental exposure contexts. Instead of ranking individual studies, we emphasize broad trends while acknowledging where methodological variability makes data interpretation challenging.

## 2 Anabaenopeptin Structure and Nomenclature

ABPs are cyclic peptides produced as secondary metabolites by cyanobacteria, commonly detected during harmful algal blooms in both freshwater and brackish environments. They have been isolated from multiple genera, including *Anabaena*, *Planktothrix*, *Oscillatoria*, *Nostoc*, and *Microcystis*.^20–25^ Biosynthetically, ABPs are assembled by nonribosomal peptide synthetases, large modular enzyme complexes capable of incorporating diverse proteogenic and nonproteogenic amino acids.^26–28^ This flexibility accounts for the high degree of structural diversity across the family.

The name “anabaenopeptin” originates from *Anabaena*, where the family was first discovered.^29^ Over time, structurally related peptides with the same macrocyclic structure have been reported under different names depending on the producing organism or collection site. Some examples include Oscillamides,^30^ Brunsvicamides,^31^ Namalides,^32^ Nodulapeptins.^33^ To simplify naming, many recent studies refer to new congeners as “Anabaenopeptin” followed by their nominal mass, for example, Anabaenopeptin 679.^34^ While this convention was intended to improve consistency, it quickly revealed its limitations as the number of reported ABPs increased. More detailed discussions are available in dedicated reviews.^35^ Table S1 summarizes ABP-type peptides and their producing organisms, providing context for the bioactivity sections that follow.

ABPs share a conserved hexapeptide scaffold in which five residues form a 19-membered macrocyclic ring and a sixth exocyclic residue (AA1) is linked to a conserved d-lysine at AA2 through a ureido bond (Figure 1). The d-lysine anchor is invariant across nearly all congeners and has been confirmed by synthetic studies, while exceptions such as the marine-derived Namalide maintain the ureido bridge despite a smaller macrocycle. Variation at the other positions introduces both chemical and functional diversity. At AA3, aliphatic residues such as Ile/*allo*-Ile or Val are the most common. AA4 frequently hosts nonproteogenic residues such as homotyrosine (Hty) or homophenylalanine (Hph), with occasional halogenated or shortened side chain variants. AA5 is almost always *N*-methylated, which increases conformational rigidity and resistance to proteolytic degradation. AA6 often features Ser, AcSer, or Met/MetO substitutions that distinguish subgroups such as nodulapeptins.^33,36^

**Figure 1:**
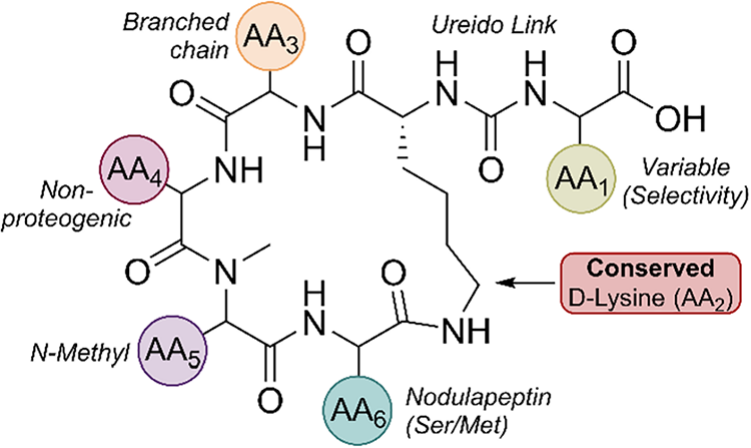
General structure of the ABPs. The scaffold consists of a conserved d-lysine (AA2) linked via a ureido bond to an exocyclic residue (AA1). AA1 is the primary determinant of metallocarboxypeptidase enzyme selectivity, while AA3 and AA6 introduce common variations: AA3 is often aliphatic, AA4 is frequently noncanonical, AA5 is usually *N*-methylated, and AA6 defines nodulapeptin subgroups (Ser/Met).

While these position-specific variations strongly influence enzyme selectivity, stereochemical assignments are not always fully resolved. Some reports suggest that the true configuration of certain congeners, including ABP F and Oscillamide Y, is l-*allo*-Ile rather than l-Ile.^37^ However, many bioactivity studies do not resolve this stereochemical detail, since the advanced Marfey’s method required to assign the β-stereocenter is often not applied.^38,39^ For this review, we present the structures as reported in the original publications, noting that residues designated as l-Ile may, in fact, be l-*allo*-Ile.

Among all positions, AA1 exerts the greatest influence on enzyme inhibition. As the solvent-exposed exocyclic residue, AA1, directly engages the enzyme active sites and largely determines the selectivity: basic residues tend to favor CPB/TAFIa inhibition. In contrast, aromatic or aliphatic residues favor CPA. Synthetic studies have exploited these variations to probe SARs, highlighting how a conserved scaffold combined with tunable peripheral residues makes ABPs an attractive platform for controlling enzyme–inhibitor interactions.

## 3 Carboxypeptidase A

CPA is a zinc-dependent exopeptidase secreted by the pancreas as an inactive zymogen and activated in the small intestine.^40^ CPA plays a central role in terminal peptide digestion by cleaving hydrophobic and aromatic residues, including phenylalanine, tyrosine, tryptophan, leucine, and isoleucine from the C-terminus of dietary peptides.^41–44^ CPA acts downstream of endopeptidases, such as trypsin and chymotrypsin, completing protein hydrolysis to yield absorbable free amino acids. Structurally, CPA belongs to the M14A subfamily of metallocarboxypeptidases and contains the conserved His–Glu–Xxx–Xxx–His zinc-binding motif.^45^ The catalytic zinc activates a water molecule for nucleophilic attack, while substrate selectivity is dictated by the S1′ pocket, a hydrophobic cleft lined by Gly207, Ile255, and Leu203 that preferentially accommodates nonpolar side chains.^46–48^

Because of its well-characterized structure, commercial availability, and predictable substrate specificity, CPA is widely employed as a model enzyme in inhibitor screening. In the context of ABPs, CPA functions as a well-established test case: modifications at the exocyclic residue (AA1) strongly influence binding affinity; changes within the macrocycle also control binding, albeit to a lesser extent. While CPA’s physiological role is limited to digestion, its well-defined active site and standardized assay conditions make the enzyme the most extensively studied ABP target, offering the clearest view of SARs. The following table (Table 1) summarizes reported IC_50_ values for natural and synthetic ABPs tested against CPA, representing the most comprehensive data set currently available for the anabaenopeptin peptide family.

**Table 1:** IC_50_ Values of Isolated Anabaenopeptins and Related Peptides against Carboxypeptidase A

	anabaenopeptin	AA1	AA2	AA3	AA4	AA5	AA6	CPA IC_50_ (μM)		refs
1	anabaenopeptin 679	NH_2_	d-Lys	l-Val	l-Hty	l-MeAla	l-Phe	6.74	^a^ ^,^ ^d^	^34^
2	anabaenopeptin 808^e^	Ile	Lys	Ile	Hty	MeAla	Phe	<30.9	^b^ ^,^ ^c^	^17^
3	anabaenopeptin 814	Phe	Lys	Val	Hty	MeGly	Phe	<4.9	^b^ ^,^ ^c^	^17^
4	anabaenopeptin 866	Ile	Lys	Val	Hph	MeHty	AcSer	40.4	^b^ ^,^ ^c^	^17^
5	anabaenopeptin 868	Ile	Lys	Val	Hph	MeHty	Met	51.8	^b^ ^,^ ^c^	^17^
6	anabaenopeptin 884	Ile	Lys	Val	Hph	MeHty	MetO	<3.4	^b^ ^,^ ^c^	^17^
7	anabaenopeptin 899	l-Phe	d-Lys	l-Val	l-Hty	l-MeHty	l-Ile	0.61	^a^ ^,^ ^d^	^49^
8	anabaenopeptin 900	Ile	Lys	Met	Hph	MeHty	Met	<31.1	^b^ ^,^ ^c^	^17^
9	anabaenopeptin 900	Phe	Lys	Val	Hph	MeHty	AcSer	<24.5	^b^ ^,^ ^c^	^17^
10	anabaenopeptin 908	l-Arg	d-Lys	l-Val	l-Hty	l-MeHty	l-Ile	>11.0	^a^ ^,^ ^d^	^34^
11	anabaenopeptin 908	l-Arg	d-Lys	l-Val	l-Hty	l-MeHty	l-Ile	>21.9	^b^ ^,^ ^d^	^50^
12	anabaenopeptin 915	l-Tyr	d-Lys	l-Val	l-Hty	l-MeHty	l-Ile	0.13	^b^ ^,^ ^d^	^50^
13	anabaenopeptin 916^e^	Phe	Lys	Val	Hty	MeHty	AcSer	<4.4	^b^ ^,^ ^c^	^17^
14	anabaenopeptin 918	Phe	Lys	Val	Hph	MeHty	MetO	<3.3	^b^ ^,^ ^c^	^17^
15	anabaenopeptin 934	Phe	Lys	Val	Hty	MeHty	MetO	21.4	^b^ ^,^ ^c^	^17^
16	anabaenopeptin A^e^	Tyr	Lys	Val	Hty	MeAla	Phe	<3.6	^b^ ^,^ ^c^	^17^
17	anabaenopeptin A^e^	Tyr	Lys	Val	Hty	MeAla	Phe	<3.6	^b^ ^,^ ^c^	^17^
18	anabaenopeptin SA6	Leu/Ile	Lys	Leu/Ile	Hph	MeAsn	Phe	4.5	^a^ ^,^ ^c^	^27^
19	anabaenopeptin 806Ne	Val	Lys	Leu/Ile	Hph	Asn	Phe	21.1	^a^ ^,^ ^c^	^27^
20	anabaenopeptin 820Ne	Val	Lys	Leu/Ile	Hph	MeAsn	Phe	3.5	^a^ ^,^ ^d^	^27^
21	anabaenopeptin B	l-Arg	d-Lys	l-Val	l-Hty	l-MeAla	l-Phe	>59.7	^b^	^6,51^
22	anabaenopeptin B	Arg	Lys	Val	Hty	MeAla	Phe	3.9	^a^ ^,^ ^d^	^7,9^
23	anabaenopeptin B	l-Arg	d-Lys	l-Val	l-Hty	l-MeAla	l-Phe	>24	^a^ ^,^ ^d^	^34^
24	anabaenopeptin C	Lys	Lys	Val	Hty	MeAla	Phe	>100.0	^a^ ^,^ ^d^	^7,9^
25	anabaenopeptin D	Phe	Lys	Val	Hty	MeAla	Phe	<3.6	^b^ ^,^ ^c^	^17^
26	anabaenopeptin E	l-Arg	d-Lys	l-Val	l-MeHty	l-MeAla	l-Phe	>58.8	^b^	^6,51^
27	anabaenopeptin F	l-Arg	d-Lys	l-Ile	l-Hty	l-MeAla	l-Phe	>24	^a^ ^,^ ^d^	^34^
28	anabaenopeptin F	l-Arg	d-Lys	l-Ile	l-Hty	l-MeAla	l-Phe	>58.8	^b^	^6,51^
29	anabaenopeptin F	Arg	Lys	Ile	Hty	MeAla	Phe	1.1	^a^ ^,^ ^d^	^7,9^
30	anabaenopeptin G	l-Tyr	d-Lys	l-Ile	l-Hty	l-MeHty	l-Ile	0.001	^b^ ^,^ ^d^	^6^
31	anabaenopeptin G	l-Tyr	d-Lys	l-Ile	l-Hty	l-MeHty	l-Ile	0.007	^b^	^51^
32	anabaenopeptin H	l-Arg	d-Lys	l-Ile	l-Hty	l-MeHty	l-Ile	10.2	^a^ ^,^ ^d^	^34^
33	anabaenopeptin H	l-Arg	d-Lys	l-Ile	l-Hty	l-MeHty	l-Ile	3.7	^b^ ^,^ ^d^	^6,51^
34	anabaenopeptin I	l-Ile	d-Lys	l-Val	l-Hty	l-MeAla	l-Leu	0.00684	^b^ ^,^ ^d^	^51^
35	anabaenopeptin J	l-Ile	d-Lys	l-Val	L-Hty	l-MeAla	l-Phe	0.00957	^b^ ^,^ ^d^	^51^
36	anabaenopeptin T	l-Ile	d-Lys	l-Val	l-Hty	l-MeHty	l-Ile	2.30	^b^ ^,^ ^d^	^6,52^
37	anabaenopeptin T	l-Ile	d-Lys	l-Val	l-Hty	l-MeHty	l-Ile	0.02540	^b^	^51^
38	namalide B	l-Ile	d-Lys	l-Ile	l-Hty			0.75	^a^ ^,^ ^d^	^53^
39	namalide C	l-Ile	d-Lys	l-Val	l-Hty			2.0	^a^ ^,^ ^d^	^53^
40	oscillamide Y	Tyr	d-Lys	Ile	Hty	MeAla	Phe	ND	^a^ ^,^ ^d^	^7^
41	oscillamide Y^e^	Tyr	Lys	Ile	Hty	MeAla	Phe	17.5	^b^ ^,^ ^c^	^17^
42	oscillamide Y	Tyr	Lys	Ile	Hty	MeAla	Phe	17.5	^b^ ^,^ ^c^	^17^

a Concentration was reported in μM.

b Concentration originally reported in μg/mL and converted to μM.

c Characterized by MSMS only.

d Characterized by NMR.

e Compound contaminated with nodularin, as reported;^17^ ND = activity was not determined or reported.

### 3.1. Inhibition of Carboxypeptidase A: Scope and Limitations

Among the biotargets of ABPs, CPA is by far the enzyme with the most published IC_50_ data. Table 1 compiles data from 11 different publications spanning the years 1999 to 2025. The IC_50_ values reported for ABP analogues against the CPA enzyme ranged from very low nanomolar levels, with ABP G (entry 30) and ABP I (entry 34) being the most potent, reported at 0.001 μM and 0.0068 μM, respectively.^6,51^ The weakest inhibitor reported was ABP C (entry 24), which had an IC_50_ greater than 100 μM.^7,9^

The CPA inhibition data in Table 1 were collected from 11 independent studies using different assay protocols and compound characterization methods. Key limitations include: (1) uncertainty in the assignment of some structures, including both residue identity and stereochemical assignment; (2) concentrations were sometimes estimated from UV absorbance with limited standards, reducing accuracy; (3) issues of sample purity, such as coisolated nodularin, occasionally made attribution difficult; and (4) variability in enzyme source, substrate, buffer composition, and detection method may have influenced potency. These methodological differences partly reflect the evolution of CPA assays, from the early use of hippuryl-l-phenylalanine with UV detection at 254 nm and long incubation times to the later use of colorimetric substrates and commercial kits. While these improvements enhance throughput, they still vary enough to make direct comparisons difficult.

### 3.2. Inhibition of Carboxypeptidase A by Isolated ABPs

Among all of the structural positions, the exocyclic residue (AA1) has the most substantial impact on CPA inhibition. Isolated ABPs with hydrophobic or aromatic residues at AA1 consistently show potent inhibition, reflecting the hydrophobic nature of CPA’s S1′ pocket. For example, ABP G (entry 30, Tyr, IC_50_ = 0.001 μM), ABP I (entry 34, Ile, IC_50_ = 0.0068 μM), and ABP 915 (entry 12, Tyr, IC_50_ = 0.13 μM) all demonstrated potent activity despite data coming from independent studies. Conversely, congeners with basic residues at AA1, such as ABP B (entry 21, Arg, IC_50_ > 59 μM) and ABP E (entry 26, Arg, IC_50_ ≥ 59 μM, inactive), were weak inhibitors.

This pattern remains consistent across various assay conditions, emphasizing the AA1 hydrophobicity as the main factor in CPA potency. Outliers, like ABP H (entry 33, Arg at AA1 but IC_50_ = 3.7 μM) versus ABP F (entry 28, Arg at AA1, IC_50_ > 59 μM) from the same study, indicate that AA1 charge alone does not fully account for CPA inhibition. Comparison of these congeners suggests that a macrocyclic context contributes to activity: ABP H contains an Ile–N-Me-Hty segment, whereas ABP F contains a Phe–N-Me-Ala segment, which has been proposed to be less favorable for a productive CPA interaction. These observations support a context-dependent interpretation of AA1 effects in which specific macrocyclic features can partially compensate for otherwise unfavorable AA1 substitutions. However, these effects remain secondary to the primary influence of AA1.

While AA1 seems to be the main factor driving CPA inhibition, residues within the macrocycle are also believed to contribute. Specifically, it was shown that ABP G (entry 30), ABP H (entry 33), and ABP T (entry 36) all contained MeHty (AA5) and Ile (AA6) motifs and each showed measurable CPA inhibition. In contrast, congeners lacking this combination, such as ABP B (entry 21), ABP E (entry 26), and ABP F (entry 28), all of which have Arg at AA1, did not inhibit CPA at concentrations up to 58 μM. ABP H (entry 33) was unusual: despite having Arg at AA1 (which is normally inactive), ABP H showed modest potency (IC_50_ = 3.7 μM). The authors suggested that this was likely due to its MeHty/Ile motif, which may have partially offset the effects of placing Arg within the S1′ pocket. Conversely, ABP G (entry 30, with Tyr at AA1) was much more potent (IC_50_ = 0.001 μM), while ABP T (entry 36, with Ile at AA1) displayed intermediate activity (IC_50_ = 2.3 μM). Based on these findings, the authors indicated that macrocyclic residues at AA5/AA6 can influence potency, but their effect remains secondary to the dominant influence of the exocyclic residue.

Overall, CPA assays offer the most comprehensive data for ABPs, serving as valuable tools for mapping SARs. The consistent patterns favoring hydrophobic and aromatic residues at the exocyclic position, along with the enhancing effects of bulky aromatics within the ring, emphasize recurring structural motifs that influence the potency. CPA inhibition data remain valuable because they demonstrate how structural differences among ABPs affect enzyme inhibition and provide a reliable system for comparing congeners. However, due to the role of CPA as a digestive enzyme, inhibition alone does not predict therapeutic or toxicological activity.

### 3.3. Inhibition of Carboxypeptidase A by Synthetic ABPs

Synthetic studies of ABPs and related peptides have provided detailed insights into the SAR for CPA inhibition. Unlike natural isolates, synthetic analogues allow systematic exploration of stereochemistry and residue contributions, leading to clear conclusions about the structural factors that influence potency. Additionally, the risk of having other cyanopeptides present as trace contaminants is also lower. Table 2 summarizes reported IC_50_ values for synthetic congeners, especially stereochemical variants of Brunsvicamide A (BVA) and structurally distinct Namalide. BVA maintains the canonical ABP scaffold with a pentacyclic 19-membered ring. In contrast, Namalide (Figure 2) has a smaller 13-membered lactam composed of three amino acids (blue) and an exocyclic phenylalanine linked (black). The chemical structures of the three synthetic Namalide variants listed in Table 2 are shown in Figure 2 for reference.

**Figure 2:**
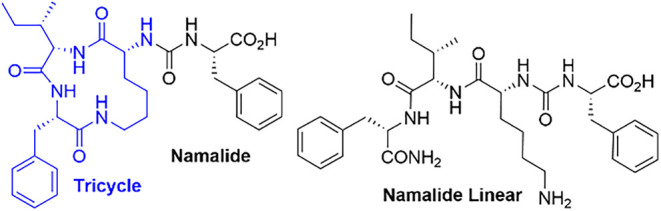
Synthetic namalide analogues evaluated for enzyme inhibition include tricycle core, linear tetrapeptide, and the natural product namalide.

**Table 2:** IC_50_ Values of Synthetic Anabaenopeptins and Related Peptides against Carboxypeptidase A

	anabaenopeptin	AA1	AA2	AA3	AA4	AA5	AA6	CPA IC_50_ (nM)	ref
1	brunsvicamide A (synthetic)	l-Ile	d-Lys	l-Val	l-Leu	l-MeTrp	l-Phe	5.0 ± 0.1	^12^
2	brunsvicamide A (derivative 1)	l-*allo*-Ile	d-Lys	l-Val	l-Leu	l-MeTrp	l-Phe	7.7 ± 0.1	^12^
3	brunsvicamide A (derivative 2)	d-*allo*-Ile	d-Lys	l-Val	l-Leu	l-MeTrp	l-Phe	17,100 ± 500	^12^
4	brunsvicamide A (derivative 3)	d-Ile	d-Lys	l-Val	l-Leu	l-MeTrp	l-Phe	27,100 ± 600	^12^
5	brunsvicamide A (derivative 4)	l-Ile	l-Lys	l-Val	l-Leu	l-MeTrp	l-Phe	5400 ± 100	^12^
6	brunsvicamide A (derivative 5)	l-*allo*-Ile	l-Lys	l-Val	l-Leu	l-MeTrp	l-Phe	>50,000	^12^
7	brunsvicamide A (derivative 6)	d-*allo*-Ile	l-Lys	l-Val	l-Leu	l-MeTrp	l-Phe	>50,000	^12^
8	brunsvicamide A (derivative 7)	d-Ile	l-Lys	l-Val	l-Leu	l-MeTrp	l-Phe	>50,000	^12^
9	brunsvicamide A (derivative 8)	l-Ala	d-Lys	l-Val	l-Leu	l-MeTrp	l-Phe	102 ± 7	^12^
10	brunsvicamide A (derivative 9)	d-Ala	d-Lys	l-Val	l-Leu	l-MeTrp	l-Phe	5700 ± 100	^12^
11	brunsvicamide A (derivative 10)	l-Ile	d-Lys	l-Val	l-Leu	l-MeTrp	l-Ala	6.6 ± 0.2	^12^
12	brunsvicamide A (derivative 11)	l-Ile	d-Lys	l-Val	l-Leu	l-MeTrp	l-Ser	7.9 ± 0.3	^12^
13	brunsvicamide A (derivative 12)	l-Ile	d-Lys	l-Val	l-Leu	l-MeAla	l-Phe	4.8 ± 0.2	^12^
14	brunsvicamide A (derivative 13)	l-Ile	d-Lys	l-Val	l-Leu	l-MeSer	l-Phe	28.9 ± 0.5	^12^
15	brunsvicamide A (derivative 14)	l-Ile	d-Lys	l-Val	l-Ala	l-MeTrp	l-Phe	8.3 ± 0.2	^12^
16	brunsvicamide A (derivative 15)	l-Ile	d-Lys	l-Val	l-Ser	l-MeTrp	l-Phe	7.6 ± 0.4	^12^
17	brunsvicamide A (derivative 16)	l-Ile	d-Lys	l-Ala	l-Leu	l-MeTrp	l-Phe	8.7 ± 0.3	^12^
18	brunsvicamide A (derivative 17)	l-Ile	d-Lys	l-Ser	l-Leu	l-MeTrp	l-Phe	22.5 ± 1.5	^12^
19	namalide (d-Lys)	l-Phe	d-Lys	l-Ile	l-Phe			250 ± 30	^32^
20	namalide (l-Lys)	l-Phe	l-Lys	l-Ile	l-Phe			>30,000	^32^
21	namalide (l-*allo*-Ile)	l-Phe	d-Lys	l-*allo*-Ile	l-Phe			^a^see notes	^32^
22	namalide linear	l-Phe	d-Lys	l-Ile	l-Phe			4500 ± 900	^32^
23	namalide tricycle		d-Lys	l-Ile	l-Phe			NA at 30,000	^32^

a For Namalide (L-*allo*-Ile), reproducibility and solubility issues were reported.

Systematic stereochemical studies on BVA confirmed the essential role of both the exocyclic Ile and the Lys configuration in CPA inhibition.^12^ Soon after the initial isolation of BVA, the configuration of the lysine residue in the macrocyclic core was reconfigured from l-Lys^54^ to d-Lys.^12^ An extensive stereochemical library was also prepared to explore the SARs. The native d-Lys, l-Ile analogue (entry 1) showed the most potent activity (IC_50_ ≈ 5 nM), while changing Lys to the l-configuration (entry 5) reduced potency to micromolar levels. In contrast, replacing l-Ile with l-*allo*-Ile (entry 2) caused a slight decrease in activity, but switching to d-Ile (entry 4) drastically reduced inhibition (IC_50_ 27,000 nM). Additional alanine and serine scans revealed that residues like Val and N-MeTrp also play significant roles: alanine substitutions generally maintained activity better than serine, indicating that nonpolar contacts are crucial. Overall, these analogues demonstrate that CPA inhibition heavily depends on the stereochemistry at Lys and the exocyclic residue, with secondary effects from macrocyclic positions.

Unlike pentacyclic BVA, studies on Namalide offered a different perspective on CPA inhibition. Namalide was synthesized along with a series of analogues to verify its stereochemistry, since limited natural material prevented direct derivatization.^32^ Their research confirmed that the natural product contains d-Lys, while all other residues are in the l-configuration. The lysine stereochemistry was crucial for bioactivity: the d-Lys analogue (entry 19) showed nanomolar inhibition (IC_50_ ≈ 250 nM), whereas the l-Lys analogue (entry 20) was inactive at concentrations below 30,000 nM. Removing the exocyclic Phe also eliminated activity, emphasizing the vital role in CPA binding. A linear analogue (entry 22) exhibited about an 18-fold decrease in potency (IC_50_ ≈ 4,500 nM). These findings underscore the significance of Lys stereochemistry, the exocyclic Phe, and the cyclic pentapeptide core in CPA inhibition, aligning with broader patterns seen across the ABPs.

Synthetic studies on BVA and Namalide demonstrate how carefully designed analogues help clarify SARs that are difficult to understand from natural isolates alone. Both series highlight the critical role of stereochemistry, particularly at the Lys residue and exocyclic position, and also emphasize the significance of exocyclic side chains in CPA inhibition. While BVA analogues focus on sensitivity to configuration at Ile and Lys, Namalide underscores the necessity of its exocyclic Phe. These findings reinforce common SAR patterns across the ABP family and show how scaffold modifications influence inhibitory profiles. Most importantly, they demonstrate that synthetic ABPs are powerful tools for studying enzyme–inhibitor interactions that surpass the capabilities of natural product mixtures.

## 4 Carboxypeptidase B (CPB)

CPB is a zinc-dependent exopeptidase secreted by the pancreas as the inactive zymogen and activated in the small intestine.^55,56^ Unlike CPA, which favors hydrophobic and aromatic side chains, CPB selectively cleaves next to basic residues, Arg and Lys, from the C-terminus of dietary peptides, thereby complementing CPA in terminal protein digestion. Structurally, CPB’s S1′ specificity pocket is lined with polar and acidic residues, including Asp255 and Ser207,^57^ which favors stabilization of positively charged side chains, in contrast to the hydrophobic pocket in CPA^58^ (Figure 3).

**Figure 3:**
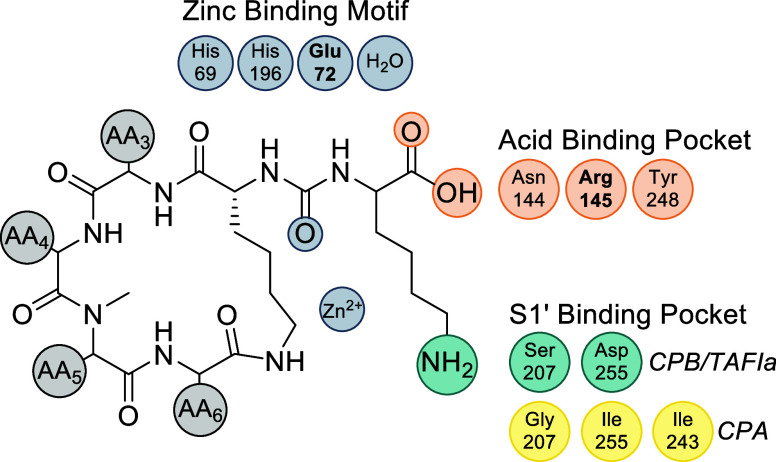
Key residue interactions between anabaenopeptin C and carboxypeptidase enzymes (CPA/CPB/TAFIa).

The preference is further exemplified in Figure 4, which illustrates ABP C docked within the active site of CPB.^9^ The exocyclic Lys establishes stabilizing interactions with Asp255 and Ser207 in the acidic S1′ pocket (Figure S1), complemented by additional hydrogen-bonding interactions involving Asn144 (2.8 Å), Arg145 (2.7 Å), and Tyr248 (3.1 Å) (Figures S2 and S3). These interactions support CPB’s strong affinity for basic residues and distinctly contrast with CPA, which preferentially binds hydrophobic substrates. For ABPs, congeners bearing Arg or Lys at the exocyclic residue (AA1) generally demonstrate enhanced binding to CPB, thereby emphasizing the differing substrate specificities of CPA and CPB. Table 3 summarizes the reported IC_50_ values for synthetic ABPs evaluated against CPB.

**Figure 4:**
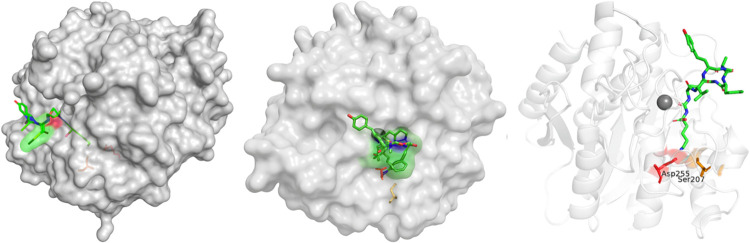
Structural views of ABP C bound to the active site of CPB (PDB 5LRJ).^9^ (Left) Side view showing ABP C (green) docked in the active site pocket. (Center) Head-on surface view illustrating the positioning of ABP C relative to the enzyme surface. (Right) Cartoon representation highlighting Asp255 and Ser207 (red and orange), key residues in the S1′ pocket that govern CPB substrate specificity. These interactions help explain the preference of CPB for basic C-terminal residues and the inhibitory activity of anabaenopeptins.

**Table 3:** IC_50_ Values of Synthetic Anabaenopeptins and Related Peptides against Carboxypeptidase B

	anabaenopeptin	AA1	AA2	AA3	AA4	AA5	AA6	CPB IC_50_ (nM)	refs
1	anabaenopeptin F (l-*allo*-Ile)	l-Arg	d-Lys	l-*allo*-Ile	l-Hty	l-MeAla	l-Phe	77^a^	^18^
2	anabaenopeptin F (l-Ile)	l-Arg	d-Lys	l-Ile	l-Hty	l-MeAla	l-Phe	61^a^	^18^
3	brunsvicamide A (synthetic)	l-Ile	d-Lys	l-Val	l-Leu	l-MeTrp	l-Phe	88 ± 006^b^	^12^
4	brunsvicamide A (derivative 1)	l-*allo*-Ile	d-Lys	l-Val	l-Leu	l-MeTrp	l-Phe	480 ± 4^b^	^12^
5	brunsvicamide A (derivative 2)	d-*allo*-Ile	d-Lys	l-Val	l-Leu	l-MeTrp	l-Phe	>50,000^b^	^12^
6	brunsvicamide A (derivative 3)	d-Ile	d-Lys	l-Val	l-Leu	l-MeTrp	l-Phe	>50,000^b^	^12^
7	brunsvicamide A (derivative 4)	l-Ile	l-Lys	l-Val	l-Leu	l-MeTrp	l-Phe	>50,000^b^	^12^
8	brunsvicamide A (derivative 5)	l-*allo*-Ile	l-Lys	l-Val	l-Leu	l-MeTrp	l-Phe	>50,000^b^	^12^
9	brunsvicamide A (derivative 6)	d-*allo*-Ile	l-Lys	l-Val	l-Leu	l-MeTrp	l-Phe	>50,000^b^	^12^
10	brunsvicamide A (derivative 7)	d-Ile	l-Lys	l-Val	l-Leu	l-MeTrp	l-Phe	>50,000^b^	^12^

a IC_50_ values determined in triplicate using porcine pancreatic CPB, Hippuryl-l-Arg substrate (1 mM), absorbance at 254 nm.

b IC_50_ values determined in triplicate using porcine pancreatic CPB, AAF-Arg-OH substrate 150 μM (≈0.6 K_M_), absorbance at 355 nm.

### 4.1. IC_50_ of Synthetic ABPs against CPB

Analogues of ABP F were synthesized to examine the stereochemical role of the isoleucine β-center.^18^ Two variants were created: one containing l-Ile (entry 2) and the other containing l-*allo*-Ile (entry 1) at AA3. Unfortunately, the spectroscopic data did not definitively assign the stereochemistry relative to the natural product. Both analogues were tested against porcine pancreatic CPB, with the l-Ile variant (entry 2) showing slightly higher potency (IC_50_ ≈ 61 nM) than the l-*allo*-Ile analogue (entry 1, IC_50_ ≈ 77 nM). These findings suggest that AA3 stereochemistry at the β-position has only a modest effect on CPB inhibition. However, the slight difference in potency should be interpreted with caution, as assay conditions may make these values the same within the range of error.^18^

In a systematic evaluation, a library of BVA analogues was synthesized and tested in a single assay format, revealing clear SAR patterns.^54^ The natural configuration of BVA (entry 3; AA1 = l-Ile, AA2 = d-Lys) inhibited CPB with an IC_50_ of 88 ± 6 nM, while the l-*allo*-Ile variant (entry 4) was less potent at 480 ± 4 nM. Exocyclic d-Ile and d-*allo*-Ile analogues (entries 5, 6) showed no activity (IC_50_ > 50,000 nM), indicating poor tolerance for d-configuration at AA1. Replacing d-Lys with l-Lys at AA2 resulted in no detectable activity (IC_50_ > 50,000 nM): all variants containing l-Lys (entries 7–10) failed to inhibit CPB at the tested concentrations. Overall, CPB inhibition with the BVA scaffold is highly dependent on the stereochemistry at both AA1 and AA2, with the d-Lys at AA2 being essential for detectable activity.

While both studies provide valuable insights, the ABP F and BVA data sets were generated in different laboratories under different assay conditions. At the same time, these synthetic efforts are crucial because they enable the evaluation of compounds with fully defined and systematically varied stereochemistry.

## 5 Thrombin-Activatable Fibrinolysis Inhibitor (TAFIa)

TAFI (also known as procarboxypeptidase U or plasma procarboxypeptidase B2) is a zinc-dependent proenzyme found in plasma and activated to TAFIa by thrombin–thrombomodulin.^59,60^ TAFIa stabilizes fibrin clots by cleaving C-terminal lysine residues from partially degraded fibrin, which reduces the binding of plasminogen and tissue plasminogen activator, downregulating fibrinolysis.^61–63^ Structurally, TAFIa belongs to the M14 family and is closely related to CPB, sharing a preference for basic residues at the S1′ pocket.^64,65^ Unlike CPB, however, TAFIa is thermally unstable, with a short half-life that significantly limits its activity *in vivo*.^66–68^ ABPs are important inhibitors because many variants contain exocyclic Arg or Lys residues at AA1 that mimic natural substrates, and several have been shown to inhibit TAFIa with low-nanomolar potency.

### 5.1. Inhibition of TAFIa by Isolated and Synthetic ABPs

Table 4 summarizes reported IC_50_ values, obtained using the American Diagnostica TAFIa assay kit, reducing the variability from results obtained using custom assays. Synthetic namalide analogues showed no measurable TAFIa inhibition up to 103,500 nM, consistent with docking results that indicated poor fit in the basic S1′ pocket due to their exocyclic Phe.^32^ In contrast, several ABPs from *Planktothrix* and *Nostoc* strains displayed potent TAFIa inhibition in the nanomolar range.^9^ The TAFIa inhibition data set shows expanded structural diversity, incorporating unusual residues such as 5-phenylnorvaline (PNV), 6-phenylnorleucine (PNL), homophenylalanine (Hph), and a chlorinated Hty (Figure 5).

**Table 4:** IC_50_ Values of Isolated Anabaenopeptins and Related Peptides against TAFIa

	anabaenopeptin	AA1	AA2	AA3	AA4	AA5	AA6	TAFIa IC_50_ (nM)		ref
1	anabaenopeptin 908	Arg	Lys	Val	Hty	MeHty	Ile	1.8	^a^ ^,^ ^c^ ^,^ ^e^	^9^
2	anabaenopeptin 915	Tyr	Lys	Val	Hty	MeHty	Ile	530	^a^ ^,^ ^c^ ^,^ ^e^	^9^
3	anabaenopeptin A	Tyr	Lys	Val	Hty	MeAla	Phe	440	^a^ ^,^ ^c^ ^,^ ^e^	^9^
4	anabaenopeptin B	Arg	Lys	Val	Hty	MeAla	Phe	1.5	^a^ ^,^ ^c^ ^,^ ^e^	^7,9^
5	anabaenopeptin C	Lys	Lys	Val	Hty	MeAla	Phe	1.9	^a^ ^,^ ^c^ ^,^ ^e^	^7,9^
6	anabaenopeptin F	Arg	Lys	Ile	Hty	MeAla	Phe	1.5	^a^ ^,^ ^c^ ^,^ ^e^	^7,9^
7	anabaenopeptin SA1	Arg	Lys	Ile	PNV	MeAsn	Phe	2.2	^a^ ^,^ ^c^ ^,^ ^e^	^9^
8	anabaenopeptin SA2	Arg	Lys	Val	Hty	MeSer	Phe	16	^a^ ^,^ ^c^ ^,^ ^e^	^9^
9	anabaenopeptin SA3	Lys	Lys	Ile	Hty	MeAla	Phe	2.1	^a^ ^,^ ^c^ ^,^ ^e^	^9^
10	anabaenopeptin SA4	Lys	Lys	Ile	PNV	MeAsn	Phe	3.4	^a^ ^,^ ^c^ ^,^ ^e^	^9^
11	anabaenopeptin SA5	Ile	Lys	Val	PNV	MeAsn	Phe	790	^a^ ^,^ ^c^ ^,^ ^e^	^9^
12	anabaenopeptin SA6	Ile	Lys	Ile	Hph	MeAsn	Phe	51,000	^a^ ^,^ ^c^ ^,^ ^e^	^9^
13	anabaenopeptin SA7	Ile	Lys	Ile	PNV	MeAsn	Phe	13,000	^a^ ^,^ ^c^ ^,^ ^e^	^9^
14	anabaenopeptin SA8	Ile	Lys	Ile	PNL	MeAsn	Phe	4800	^a^ ^,^ ^c^ ^,^ ^e^	^9^
15	anabaenopeptin SA9	Phe	Lys	Ile	Cl-Hty	MeGly	Hph	31,000	^a^ ^,^ ^c^ ^,^ ^e^	^9^
16	anabaenopeptin SA10	Phe	Lys	Val	Hty	MeGly	Cl-Hty	7000	^a^ ^,^ ^c^ ^,^ ^e^	^9^
17	anabaenopeptin SA11	Phe	Lys	Ile	Hty	MeGly	Cl-Hty	15,000	^a^ ^,^ ^c^ ^,^ ^e^	^9^
18	anabaenopeptin SA12	Phe	Lys	Val	Hty	MeGly	Hty	4300	^a^ ^,^ ^c^ ^,^ ^e^	^9^
19	anabaenopeptin SA13	Tyr	Lys	Val	Hty	MeSer	Phe	2500	^a^ ^,^ ^c^ ^,^ ^e^	^9^
20	namalide (d-Lys)	l-Phe	d-Lys	l-Ile	l-Phe			>103,500	^b^ ^,^ ^d^	^32^
21	namalide (l-Lys)	l-Phe	l-Lys	l-Ile	l-Phe			>103,500	^b^ ^,^ ^d^	^32^
22	namalide (linear)	l-Phe	d-Lys	l-Ile	l-Phe			>103,500	^b^ ^,^ ^d^	^32^
23	namalide tricycle		d-Lys	l-Ile	l-Phe			>103,500	^b^ ^,^ ^d^	^32^
24	oscillamide Y	Tyr	Lys	Ile	Hty	MeAla	Phe	400	^a^ ^,^ ^c^ ^,^ ^e^	^7,9^

a Concentration was reported in nM.

b Concentration was reported in μM and converted to nM.

c Isolated from cultured strains of cyanobacteria.

d Synthetic analogues with known stereochemistry.

e Characterized by NMR. IC_50_ values were determined using the Actichrome Plasma TAFIa activity kit (American Diagnostica), which allows for greater cross-study comparability. The prefix “Me-” denotes *N*-methylation at AA5. Abbreviations: Hty, homotyrosine; Cl-Hty, 2-chloro-homotyrosine; Hph, homophenylalanine; PNV, 5-phenylnorvaline; PNL, 6-phenylnorleucine.

**Figure 5:**
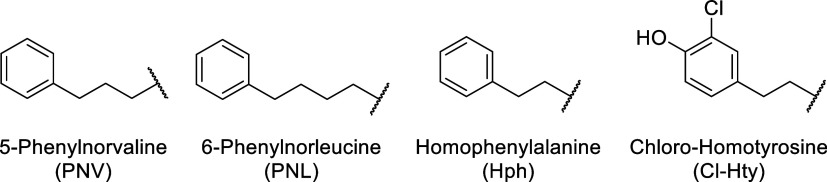
Structures of selected nonproteinogenic amino acids identified in isolated anabaenopeptins evaluated in Table 4.

### 5.2. TAFIa IC_50_ Data SAR Trends

SAR trends in Table 4 highlight the key role of the exocyclic residue (AA1). Peptides with basic residues (Arg or Lys) at this position consistently show low-nanomolar potency, with IC_50_ values from 1.5 nM (ABP B, entry 4; ABP F, entry 6) to 16 nM (ABP SA2, entry 8). Replacing these basic side chains with aromatic groups causes significant activity loss: Tyr decreases potency by about 2 orders of magnitude, with IC_50_ values of 400 nM for Oscillamide Y (entry 24) and 530 nM for ABP 915 (entry 2). Replacement with Phe or Ile resulted in weaker activity, yielding a micromolar potency. For example, ABP SA6 (entry 12, Ile at AA1) inhibited with an IC_50_ of 51,000 nM, while ABP SA11 (entry 17, Phe at AA1) showed an IC_50_ of 15,000 nM. This trend in AA1 potency has been summarized as Arg = Lys ≫ Tyr > Phe ≈ Ile.^9^

At other positions, more nuanced SAR patterns appeared. Substitution at AA5 was somewhat restrictive: MeAla was preferred, while adding MeSer caused a 5–10-fold decrease in potency. For instance, within a single study, most congeners with Arg or Lys at the exocyclic position exhibited IC_50_ values of 1.5–2.2 nM, whereas ABP SA2 (entry 8) was less potent (16 nM). ABP SA2 differs from ABP B (entry 4) only at AA5, where MeSer replaces MeAla, reducing potency from 1.5 to 16 nM. A comparable effect was seen when comparing congeners with tyrosine in the exocyclic position, ABP A (entry 3) and ABP SA13 (entry 13), where the MeAla to MeSer substitution at AA5 reduced potency from 440 nM to 2500 nM.^9^ While substitutions at AA5 are secondary to AA1, they are more influential than at other positions

Positions AA4 and AA6 were more adaptable, accepting different aromatic and aliphatic residues with only slight changes in potency when paired with complementary substitutions at AA5. For example, ABP F (entry 6; Hty/MeAla at AA4/AA5) and ABP SA1 (entry 7; PNV/MeAsn) inhibited with nearly identical IC_50_ values (1.5 vs 2.2 nM), and a similar outcome was observed for ABP SA3 (entry 9; 2.1 nM) and ABP SA4 (entry 10; 3.4 nM). At AA6, aliphatic substitutions were likewise well tolerated: ABP F (entry 6; MeAla/Phe) inhibited at 1.5 nM, while ABP 908 (entry 1; MeHty/Ile) maintained comparable potency at 1.8 nM.

In comparison, AA3 substitutions contributed even less to the observed TAFIa SAR trends, with Val and Ile behaving as interchangeable residues. ABP B (entry 4, Val) and ABP F (entry 6, Ile) both inhibited with IC_50_ values of 1.5 nM, and a similar pattern was observed for ABP C (entry 5, Val, 1.9 nM) and ABP SA3 (entry 9, Ile, 2.1 nM)

The overall impact of each residue position is summarized in Figure 6. AA1 dominates potency trends, serving as the primary driver of the activity. AA5 is secondary, while not as critical as AA1, and certain substitutions (N-Me Ala/Ser) have a reproducible and substantial effect on potency. AA4 and AA6 are more permissive, tolerating a range of aromatic and aliphatic residues. Finally, AA3 has a minimal impact, as Val and Ile substitutions are functionally interchangeable. Overall, this hierarchy can be described as AA1 ≫ AA5 > AA4/AA6 ≈ AA3. This pattern contrasts with CPA, where aromatic and aliphatic residues are preferred at the exocyclic position, underscoring how slight differences in the S1′ pocket environment translate into divergent selectivity profiles, whereas substitutions within the peptide macrocycles are more tolerated.

**Figure 6:**
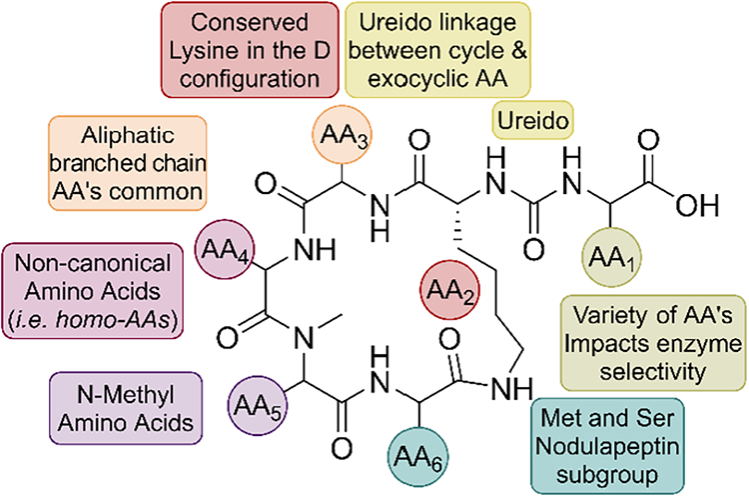
Structure–activity relationship trends of anabaenopeptins against TAFIa. The exocyclic residue (AA1) drives potency: Arg/Lys confers nanomolar inhibition, Tyr intermediate, and weak Phe/Ile activity. Val/Ile are interchangeable at AA3, AA2 (d-Lys), and the ureido linkage is conserved, multiple aromatics are tolerated at AA4/AA6, and MeAla is favored over MeSer at AA5 (5–10× difference).

## 6 Protein Phosphatase 1

Protein Phosphatase 1 (PP1) is a serine/threonine phosphatase of the PPP family that regulates diverse cellular processes such as glycogen metabolism, cell cycle progression, muscle contraction, and synaptic plasticity.^69–74^ PP1 functions by dephosphorylating phosphoserine and phosphothreonine residues, thereby reversing kinase-driven signaling and maintaining the phosphorylation balance. Structurally, PP1 contains a conserved catalytic domain with metal-binding motifs (GDxHG, GDxVDRG, GNHE) that coordinate a binuclear metal center (metal pairs can be any combination of these, usually Mn^2+^/Fe^2+^, though other divalent cations can substitute), essential for catalysis.^75,76^ Substrate specificity and localization are dictated not by the catalytic core alone but by association with more than 200 regulatory proteins, which guide PP1 to distinct compartments and substrates.

From a biomedical perspective, PP1 activity is critical in pathways related to cancer, neurodegeneration, cardiac function, and toxin sensitivity. Dysregulated PP1 signaling is implicated in abnormal cell growth and memory impairment, and selective inhibitors such as okadaic acid, microcystins, and tautomycetin serve as both biochemical probes and toxicological benchmarks, typically exhibiting IC_50_ values in the low nanomolar range. Inhibition studies are often conducted using colorimetric or fluorometric substrates (e.g., pNPP, DiFMUP) or phosphopeptides, though assay variability, including metal cofactors and substrate choice, can complicate direct comparisons of IC_50_ values. This context underscores the significance of evaluating PP1 inhibition by ABPs and related cyclic peptides, whose ureido-linked exocyclic residues are proposed to engage the catalytic site and block catalytic turnover.

## 7 Protein Phosphatase 2A

Protein Phosphatase 2A (PP2A) is another major serine/threonine phosphatase of the PPP family and one of the most abundant phosphatases in eukaryotic cells.^77,78^ PP2A regulates critical processes, including cell growth, signal transduction, apoptosis, and cytoskeletal dynamics, and is frequently regarded as a tumor suppressor. PP2A functions by dephosphorylating serine and threonine residues in pathways including PI3K/AKT, MAPK, and Wnt.^79,80^ Structurally, PP2A is typically assembled as a heterotrimeric holoenzyme comprising a catalytic C subunit, a scaffolding A subunit, and one of several B subunits that confer substrate selectivity and localization. Like PP1, the catalytic core contains the conserved PPP motifs (GDxHG, GDxVDRG, GNHE) that coordinate a binuclear Mn^2+^/Fe^2+^ center, which is essential for activity.

From a biomedical perspective, dysregulated PP2A activity is linked to cancer, neurodegeneration, and viral infections, and pharmacological inhibition or reactivation holds therapeutic potential.^81–84^ PP2A is strongly inhibited by natural compounds such as okadaic acid, microcystins, and calyculin A, with IC_50_ values in the picomolar to low nanomolar range, making PP2A more sensitive to these toxins than PP1.^85–87^ Assays typically use pNPP or phosphopeptide substrates, requiring careful distinction to differentiate PP2A inhibition from PP1 inhibition due to their overlapping sensitivities. This biochemical context offers a foundation for assessing inhibition by ABPs and related peptides, which may target the conserved catalytic site but could also be influenced by the unique regulatory structure of PP2A holoenzymes.

### 7.1. IC_50_ Data Overview and General Trends

The available inhibition data for ABPs against protein phosphatases are summarized in Tables 5–6. For PP1 (Table 5), reported IC_50_ values range from the low nanomolar (ABP 934, 17 nM, entry 11)^17^ to the tens of micromolar (ABP F, 28,200 nM, entry 18).^19^ Compared to the extensive data set available for carboxypeptidases, phosphatase data remain limited, with most values coming from only three publications.

**Table 5:** IC_50_ Values of Isolated Anabaenopeptins and Related Peptides against Protein Phosphatase 1 (PP1)^a^

	anabaenopeptin	AA1	AA2	AA3	AA4	AA5	AA6	PP1 IC_50_ (nM)	% Inh. PP1		refs
1	anabaenopeptin 808^a^	Ile	Lys	Ile	Hty	MeAla	Phe	68		^b^ ^,^ ^c^	^17^
2	anabaenopeptin 814	Phe	Lys	Val	Hty	MeGly	Phe	534		^b^ ^,^ ^c^	^17^
3	anabaenopeptin 866	Ile	Lys	Val	Hph	MeHty	AcSer	503		^b^ ^,^ ^c^	^17^
4	anabaenopeptin 868	Ile	Lys	Val	Hph	MeHty	Met	82		^b^ ^,^ ^c^	^17^
5	anabaenopeptin 870	Phe	Lys	Val	Leu	MeHty	MetO	76		^b^ ^,^ ^c^	^17^
6	anabaenopeptin 884	Ile	Lys	Val	Hph	MeHty	MetO	60		^b^ ^,^ ^c^	^17^
7	anabaenopeptin 900	Ile	Lys	Met	Hph	MeHty	Met	67		^b^ ^,^ ^c^	^17^
8	anabaenopeptin 900	Phe	Lys	Val	Hph	MeHty	AcSer	156		^b^ ^,^ ^c^	^17^
9	anabaenopeptin 916^a^	Phe	Lys	Val	Hty	MeHty	AcSer	55		^b^ ^,^ ^c^	^17^
10	anabaenopeptin 918	Phe	Lys	Val	Hph	MeHty	MetO	109		^b^ ^,^ ^c^	^17^
11	anabaenopeptin 934	Phe	Lys	Val	Hty	MeHty	MetO	17		^b^ ^,^ ^c^	^17^
12	anabaenopeptin A^a^	Tyr	Lys	Val	Hty	MeAla	Phe	104		^b^ ^,^ ^c^	^17^
13	anabaenopeptin A^a^	Tyr	Lys	Val	Hty	MeAla	Phe	102		^b^ ^,^ ^c^	^17^
14	anabaenopeptin A	Tyr	Lys	Val	Hty	MeAla	Phe		40–60%	^d^	^88^
15	anabaenopeptin B	Arg	Lys	Val	Hty	MeAla	Phe		5–75%	^d^	^88^
16	anabaenopeptin B	Arg	Lys	Val	Hty	MeAla	Phe	9500 ± 900		^a^	^19^
17	anabaenopeptin D	Phe	Lys	Val	Hty	MeAla	Phe	64		^b^ ^,^ ^c^	^17^
18	anabaenopeptin F	Arg	Lys	Ile	Hty	MeAla	Phe	28,200 ± 3400			^19^
19	anabaenopeptin F	l-Arg	d-Lys	l-Ile	l-Hty	l-MeAla	l-Phe		38.10%	^e^	^13^
20	oscillamide B	l-Arg	d-Lys	l-Met	l-Hty	l-MeAla	l-Phe		43.30%	^e^	^13^
21	oscillamide C	l-Arg	d-Lys	l-Ile	l-Hty	l-MeHty	l-Phe	900	97.30%	^e^	^13^
22	oscillamide Y^a^	Tyr	Lys	Ile	Hty	MeAla	Phe	72		^b^ ^,^ ^c^	^17^
23	oscillamide Y	Tyr	Lys	Ile	Hty	MeAla	Phe	72		^b^ ^,^ ^c^	^17^
24	oscillamide Y	l-Tyr	d-Lys	l-Ile	l-Hty	l-MeAla	l-Phe		11.20%	^e^	^13^

a Compound contaminated with nodularin, as reported.^17^

b Concentration originally reported in μg/mL and converted to nM.

c Characterized by MSMS only.

d Activity reported as % inhibition of PP1 (0.009–4.5 μg/mL), not IC_50_.

e Activity reported only as % inhibition of PP1 at 100 μg/mL; IC_50_ not determined.

**Table 6:** IC_50_ and % Inhibition Values of Anabaenopeptins and Related Peptides against Protein Phosphatases 2A^c^,^e^

	anabaenopeptin	AA1	AA2	AA3	AA4	AA5	AA6	PP2A IC_50_ (nM)	% Inh. PP2A		refs
25	anabaenopeptin B	Arg	Lys	Val	Hty	MeAla	Phe	12,300 ± 6100		^a^	^19^
26	anabaenopeptin F	Arg	Lys	Ile	Hty	MeAla	Phe	1000 ± 200		^a^	^19^
27	anabaenopeptin F	l-Arg	d-Lys	l-Ile	l-Hty	l-MeAla	l-Phe		41.50%	^d^ ^,^ ^f^	^13^
28	oscillamide B	l-Arg	d-Lys	l-Met	l-Hty	l-MeAla	l-Phe		62.80%	^d^ ^,^ ^f^	^13^
29	oscillamide C	l-Arg	d-Lys	l-Ile	l-Hty	l-MeHty	l-Phe	133	98.40%	^a^ ^,^ ^b^	^13^
30	oscillamide Y	l-Tyr	d-Lys	l-Ile	l-Hty	l-MeAla	l-Phe		9.60%	^f^	^13^

a Concentration was reported in μM.

b Concentration originally reported in μg/mL and converted to nM.

c Characterized by MSMS only.

d Characterized by NMR.

e Activity reported as % inhibition of PP1 (0.009–4.5 μg/mL), not IC_50_.

f Activity reported only as % inhibition of PP1 at 100 μg/mL; IC_50_ not determined.

However, some clear patterns are evident: some reports consistently find IC_50_ values in the sub- to low-nanomolar range,^17^ whereas others observe only micromolar inhibition under their assay conditions.^19^ PP2A inhibition by ABPs (Table 6) has been examined far less extensively than PP1, with only a few data sets available.^13,19^ Therefore, PP2A data are considered here alongside PP1 data to highlight the shared SAR rather than to suggest an entirely separate body of evidence.

### 7.2. PP1 and PP2A Inhibition by ABPs (Tables 5–6)

The earliest systematic SAR analysis involving ABPs and phosphatase enzymes examined four congeners: ABP F (entry 19/27), oscillamide B (entry 20/28), oscillamide C (entry 21/29), and oscillamide Y (entry 24/30).^13^ Oscillamide C (AA1 = Arg) was the most potent, inhibiting PP1 with an IC_50_ of 900 nM and showing 97.3% inhibition at 105 μM (Table 5, entry 21); PP2A potency was similar (IC_50_ = 133 nM; 98.4% at 105 μM; Table 6, entry 29). Oscillamide B (entry 20) and ABP F (entry 19) (both AA1 = Arg) were much weaker (43% inhibition at 115 μM and 38.1% inhibition at 117.5 μM; Table 5); however, they were far more potent than oscillamide Y (Table 5, entry 24) (AA1 = Tyr), which was nearly inactive (11.2% inhibition at 117 μM). These results led to the conclusion that the exocyclic ureido-linked Arg residue was crucial for activity. Additionally, the potency could be further increased when the AA5 residue features a bulky hydrophobic character, as observed with MeHty instead of MeAla.

Soon after, inhibition of PP1 by ABP A (entry 14) and ABP B (entry 15) was reported, with ABP A (AA1 = Tyr) showing 40–60% inhibition and ABP B (AA1 = Arg) showing 5–75% inhibition when tested at 0.01–5.3 μM.^88^ The results aligned with earlier findings, indicating that the arginine side chain plays a more significant role in inhibition, as observed with ABP B. In contrast, tyrosine is less effective, as demonstrated with ABP A.^13^

This work was later expanded to the most comprehensive data set on PP1 inhibition by ABP-type peptides, which included 14 congeners, several of which belong to the nodulapeptins subgroups, as indicated by the presence of Met and Ser in their sequences (Table 5).^17^ All congeners inhibited PP1, with IC_50_ values ranging from 17 to 534 nM. Importantly, potent inhibition was observed even for non-Arg analogues: ABP 934 (AA1 = Phe; IC_50_ = 17 nM), ABP 884 (AA1 = Ile; IC_50_ = 60 nM). Although it may seem that this study contradicts the earlier conclusion that Arg was strictly required, these differences in IC_50_ values could be attributed to different assay conditions. Another feature presented in Table 5 is the reported inhibition of oscillamide Y. Oscillamide Y was isolated as a pure fraction (entry 23) and as a contaminated fraction (entry 22) with 9 ng/mL of nodularin detected, which appears not to impact PP1 inhibition, as both showed the same inhibition activity toward PP1 (IC_50_ = 72 nM). Nodularin is a well-known potent PP1 inhibitor with hepatotoxic capabilities.^89,90^

More recently, the PP1 inhibition activity of ABP B (entry 16) and F (entry 18) (Table 5) (both AA1 = Arg) was reported with IC_50_ values of 9,500 nM and 28,200 nM, respectively.^19^ The same work also described PP2A inhibition (Table 6), with IC_50_ values of 12,300 nM for ABP B (entry 25) and 1,000 nM for ABP F (entry 26).^19^ The only difference between ABP B and ABP F is the residue at AA3 within the macrocycle core, where ABP B has a valine, and ABP F has an isoleucine. ABP F showed potent activity toward PP2A (Table 6) and lower activity toward PP1 (Table 5), while ABP B showed opposing data, potent activity toward PP1 and lower activity toward PP2A.^19^ Additional studies have examined ABP activity against PP1 and PP2A; however, because the reported values were derived from mixed fractions, they were excluded from the tables to avoid confusion.^91^

Taken together, these studies indicate that while Arg-containing ABPs can inhibit both PP1 and PP2A, potency is highly variable and scaffold-dependent. Early SAR emphasized the importance of Arg and N-MeHty. Still, later findings demonstrated that strong inhibition can also arise from non-Arg congeners, while some Arg-containing peptides remain weak inhibitors. These results underscore the complexity of ABP-phosphatase interactions and highlight the need for broader, standardized evaluations.

### 7.3. Potential Implications of PP1/PP2A Inhibition

Although the inhibitory properties of ABPs for PP1/PP2A are modest compared with those of metallocarboxypeptidases, these studies have yielded additional insights. Discrepancies between phosphatase-based assays in fish tissues during microcystin investigations revealed findings that ABPs and/or ABP-related compounds were detected in aquatic animal tissues at concentrations exceeding those measured in the bloom waters.^88,92^ Therefore, not only are these compounds present in water sources and possess the ability to disrupt phosphatase activity, but they are also being taken up into animal tissues, including fish that humans consume. Furthermore, these data suggest the occurrence of bioaccumulation since they are present in higher concentrations in the aquatic animal tissue than in the surrounding waters, which not only is a pressing concern of ecological importance but also poses a potential human health threat relevant to those who consume fish. Taking these facts into consideration, other studies show that ABP B and ABP F disrupt cytoskeletal organization in astrocytes, and that ABP B can insert into lipid bilayers, suggesting that some congeners may not require transport to influence intracellular processes.^19^

### 7.4. Potential Implications of TAFIa Inhibition

While the inhibitory activity of ABPs against TAFIa is well established *in vitro*, the physiological effects of environmental exposure remain uncertain. A speculative but important concern is that ABPs in contaminated waters could be absorbed through the skin, particularly in individuals with compromised skin barriers, such as those with eczema or open wounds.^93,94^ People with active eczema lesions often have open or weeping sores, raising the question of whether bioactive compounds such as ABPs might penetrate the skin and interfere with local physiological processes, including wound healing.

TAFIa plays a key role in controlling fibrinolysis during tissue repair. Once activated, TAFIa cleaves C-terminal lysine residues from partially broken-down fibrin, which decreases plasminogen and tissue plasminogen activator binding to the fibrin surface. This reduces plasmin formation and stabilizes the forming clot, preventing early dissolution and aiding effective tissue repair. Inhibiting TAFIa with external molecules such as ABPs, especially under localized inflammatory conditions, could disrupt this balance and hinder healing. To date, no studies have specifically examined the impact of natural TAFIa inhibitors, such as ABPs, on skin healing or their activity in the dermal environment. This gap in understanding highlights a potential connection between environmental toxicology and human health, which warrants further investigation.

### 7.5. Potential Implications of CPA and CPB Inhibition

Exposure to ABPs through drinking water or ingestion may also have health implications. One hypothesis is that accidental ingestion of CPA and/or CPB inhibitors would have little effect since most peptides are broken down in the gut, and protein digestion is aided by multiple enzymes. However, ABPs are unusually chemically stable; they require extreme conditions (6 M HCl, 110 °C, 24 h) to be completely hydrolyzed in laboratory tests. Compared to these harsh conditions, physiological gastric conditions, dilute hydrochloric acid at 37 °C, are much milder, indicating that ABPs are unlikely to be fully broken down during digestion.^95^ As a result, intact peptides or large fragments could reach the intestine, where they might inhibit pancreatic CPA and CPB. Repeated or chronic ingestion, such as during harmful algal blooms, could suppress CPA and CPB activity in the gut lumen, reducing the protein digestion efficiency. The persistent longer peptides would increase the flow of undigested substrates to the colon, where microbial fermentation produces ammonia, hydrogen sulfide, phenols, and indoles, metabolites associated with mucosal irritation, oxidative stress, and compromised barrier function. Over time, these conditions could lead to a dysbiotic microbiome enriched with proteolytic species, potentially causing inflammation or altering host–microbe interactions.^96^

Thus, even if systemic absorption of ABPs is minimal, their chemical resilience and inhibitory activity suggest that environmental exposure could impact gut microbial ecology and intestinal health. Currently, no published studies examine the fate of orally ingested ABPs in mammals or their potential to modify gut microbial communities or peptide metabolism. While similar evidence exists for other cyanobacterial toxins, such as microcystin-LR, which alters microbiome composition in rodent models,^97,98^ this gap highlights the need for further research.^93,99^ Given ABPs’ stability and ability to inhibit digestive enzymes, this area presents a compelling opportunity for future study at the crossroads of environmental toxicology, microbiome science, and human health.

### 7.6. Comparison to Benchmark Inhibitors

At present, there is no universally adopted assay kit or standardized protocol for evaluating ABP inhibition across enzyme targets; however, the use of benchmark inhibitors represents a practical best-practice approach to improve comparability across studies. A significant gap in the current literature is the lack of direct comparisons between the IC_50_ values of ABPs and those of known benchmark inhibitors of the same enzymes. In many enzyme inhibition studies, a well-characterized reference inhibitor, such as benzylsuccinic acid for CPA, is often used as a standard to provide a consistent point of reference. This enables easier comparison of potency across different studies and experimental conditions. However, in the case of ABPs, none of the reviewed studies included such a benchmark comparison. Consequently, the IC_50_ values reported for CPA, CPB, TAFIa, and PP1 and PP2A remain study-dependent. While these values are useful on their own, the absence of a common reference point means that direct cross-study comparisons should be approached with caution.^100^ Future studies may benefit from including a known benchmark inhibitor in their assays to establish a clearer baseline. This would not only allow for more accurate comparisons of ABP potency but would also offer greater insight into their relative effectiveness as enzyme inhibitors.

## 8. Conclusion

ABPs and related cyanobacterial peptides are a chemically diverse and biologically active class of natural products. Their capacity to inhibit various enzyme families, especially carboxypeptidases A and B, TAFIa, and the serine/threonine phosphatases PP1 and PP2A, reflects their structural flexibility and increasing importance in both environmental and pharmacological areas. This review compiles over 20 years of IC_50_ data to highlight key SARs, notably the strong influence of the exocyclic residue (AA1) and the adjustable roles of macrocyclic residues. For all targets studied, basic residues at AA1 promote TAFIa inhibition, while hydrophobic residues favor CPA binding. The inhibition of phosphatase activity varies, with both Arg-containing and non-Arg-containing analogues showing activity. Synthetic research has also emphasized the significance of stereochemistry, exocyclic side chains, and macrocycle structure in determining potency and selectivity.

However, this analysis also reveals substantial limitations in the current body of data. Many studies lack benchmark comparisons, differ widely in assay design, and report IC_50_ values without consistent units or structural validation. These inconsistencies hinder cross-study comparisons and obscure genuine SAR trends. To move forward, future research should prioritize standardized assay protocols, verified structural assignments, and the inclusion of known reference inhibitors.

Beyond biochemical assays, this Perspective also considers the potential toxicological and physiological implications of ABP exposure. Due to their unusual chemical stability, ABPs may persist through digestion and inhibit digestive enzymes in the gut lumen, potentially leading to downstream effects on microbiome composition and intestinal health. Their potent inhibition of TAFIa raises questions about the impact on wound healing, particularly in individuals with compromised skin barriers. While intracellular access remains uncertain, recent studies suggest that some ABPs can insert into lipid membranes and disrupt cytoskeletal organization in mammalian cells, providing a plausible mechanism for phosphatase inhibition in nonhepatic tissues.^19^

Together, these findings highlight the dual nature of ABPs: as valuable tools for dissecting enzyme function and as potential environmental toxins with systemic effects. ABPs’ structural diversity, biological potency, and ecological occurrence position them as compelling candidates for further exploration in chemical biology, toxicology, and drug discovery. With more rigorous and standardized methodologies, the full biological and translational potentials of these complex peptides can be more clearly realized.

## Supplementary Material





## Notes

Structural models of APB C bound to CPB, comparison of conserved CPA/CPB active-site features, a catalog of anabaenopeptin natural products and source organisms, standardized IC_50_ data sets for CPA, PP1, and PP2A with notes on stereochemical variants and activity trends (PDF)
SMILES data for ABP variants (XLSX)

## Abbreviations

ABPs: anabaenopeptins
IC_50_: half-maximal inhibitory concentration
SARs: structure–activity relationships
MS/MS: tandem mass spectrometry
CPA: carboxypeptidase A
CPB: carboxypeptidase B
TAFI: thrombin-activatable fibrinolysis inhibitor
TAFIa: activated form of TAFI
PP1: protein phosphatase 1
PP2A: protein phosphatase 2A
BVA: brunsvicamide A
Hty: homotyrosine
Hph: homophenylalanine
Cl-Hty: 2-chloro-homotyrosine
PNV: 5-phenylnorvaline
PNL: 6-phenylnorleucine
MeAla: *N*-methylalanine
MeGly: *N*-methylglycine
MeSer: *N*-methylserine
MeTrp: *N*-methyltryptophan
MeHty: *N*-methylhomotyrosine
MeAsn: *N*-methylasparagine
AcSer: O-acetylserine
MetO: methionine sulfoxide
Glu–Xxx–Xxx–His: conserved zinc-binding motif
PPP: phosphoprotein phosphatase family
GDxHG/GDxVDRG/GNHE: conserved metal-binding motifs
pNPP: para-nitrophenyl phosphate
DiFMUP: 6,8-difluoro-4-methylumbelliferyl phosphate
PI3K: phosphoinositide 3-kinase
Akt: protein kinase B
MAPK: mitogen-activated protein kinase
Wnt: wingless-related integration site signaling pathway
