# Supplementary material for: Structure–Activity Relationships of Anabaenopeptins as Carboxypeptidase and Phosphatase Inhibitors
Source: ACS Chem Biol. 2026 Jan 21;21(2):210–23. doi: 10.1021/acschembio.5c00791 (PMC12927552; doi:10.1021/acschembio.5c00791)
Supplement: Supplementary file 1 [file cb5c00791_si_001.pdf]

Supporting Information

Structure–Activity Relationships of Anabaenopeptins as Carboxypeptidase and Phosphatase Inhibitors

Megan L. Quandt, Judy Westrick, Jeremy J. Kodanko*

Department of Chemistry, Wayne State University, 5101 Cass Avenue, Detroit, MI 48202, USA.

Table of Contents

Figure S1. Recognition of the Lysine Side Chain of ABP C in the CPB S1′ Pocket.

Figure S2. Recognition of ABP C Across the S1′ and Acid-Binding Pockets of CPB.

Figure S3. Recognition of ABP C in the CPB Acid-Binding Pocket.

Figure S4. Overlay of CPA and CPB Active Sites Highlighting Conserved Active Site Residues.

Table S1. Anabaenopeptin Type Natural Products and Source Organisms.

Table S2. IC_{50} Values of Anabaenopeptins Against CPA (Converted to Molar Units).

Table S3. IC_{50} Values of Anabaenopeptins Against PP1 (Converted to Molar Units).

Table S4. IC_{50} Values and % Inhibition of PP2A by Anabaenopeptins (Converted to Molar Units).

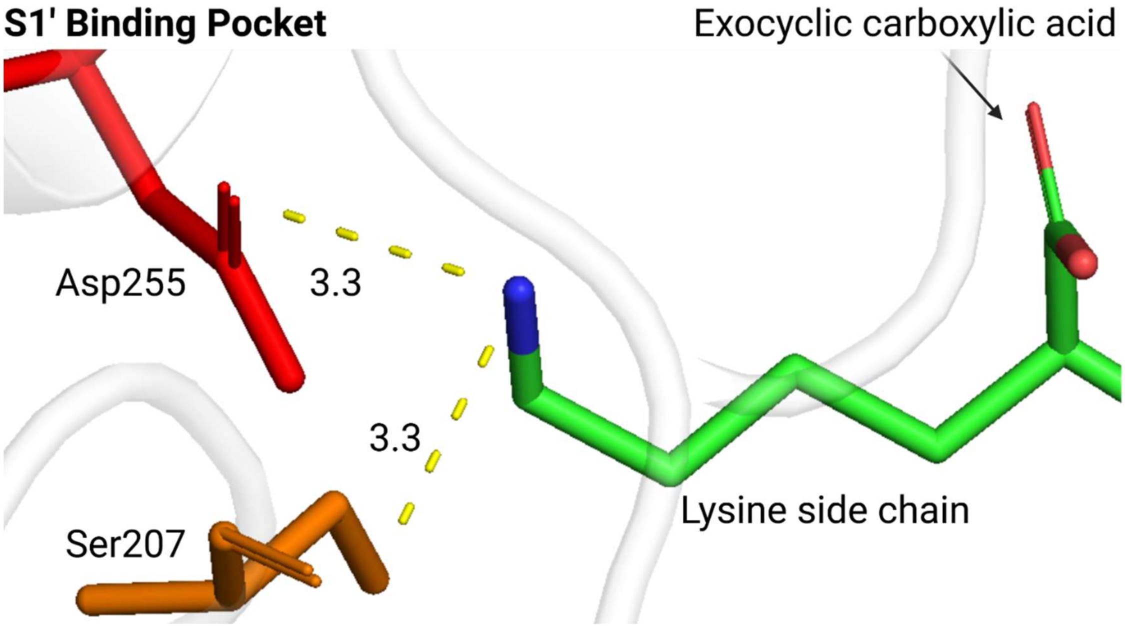

Figure S1: Recognition of the Lysine Side Chain of ABP C in the CPB S1′ Pocket. Interaction of ABP C with the S1′ pocket of CPB (PDB 5LRJ)[1]. The exocyclic lysine side chain (green) forms hydrogen bond contacts (3.3 Å) with Asp255 (red) and Ser207 (orange), residues that govern substrate specificity in CPB.

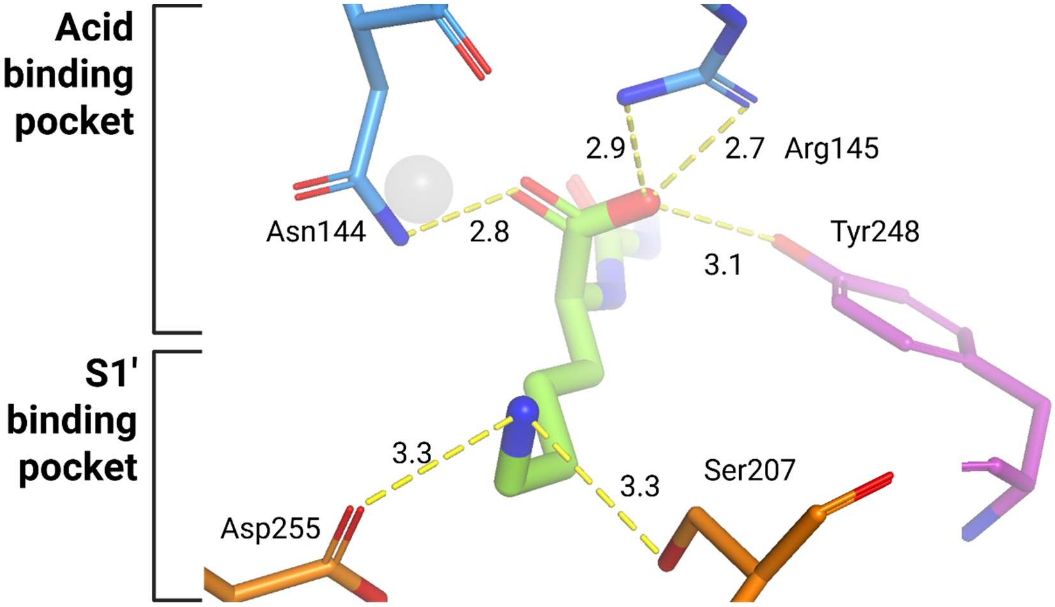

Figure S2. Recognition of ABP C Across the S1′ and Acid-Binding Pockets of CPB. Interaction of ABP C with the active site of carboxypeptidase B (CPB, PDB 5LRJ)[1]. The exocyclic lysine side chain (green) engages the S1′ binding pocket through hydrogen bonds with Asp255 (3.3 Å, orange) and Ser207 (3.3 Å, orange), residues that define substrate specificity in CPB. At the same time, the terminal carboxylate interacts with the acid-binding pocket, forming hydrogen bonds with Asn144 (2.8 Å, blue), Arg145 (2.7 Å, blue), and the phenolic hydroxyl of Tyr248 (3.1 Å, pink). Together, these contacts show how ABP C bridges the S1′ and acid-binding pockets, stabilizing inhibitor binding within the CPB active site.

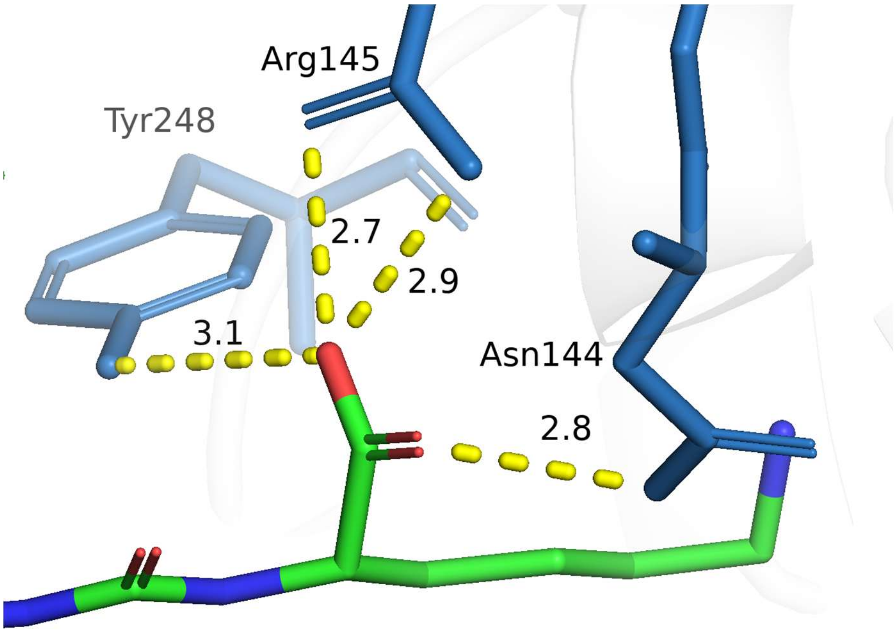

Figure S3. Recognition of ABP C in the CPB Acid-Binding Pocket. Close-up of ABP C in the acid-binding pocket of carboxypeptidase B (CPB)(PDB 5LRJ)[1]. The exocyclic carboxylic acid (green) forms hydrogen bonds with Arg145 (2.7 and 2.9 Å, dark blue), the hydroxyl of Tyr248 (3.1 Å, light blue), and the carbonyl of Asn144 (2.8 Å, light blue). These interactions emphasize the role of the acid-binding pocket in recognizing and stabilizing the terminal carboxylate group of ABP C.

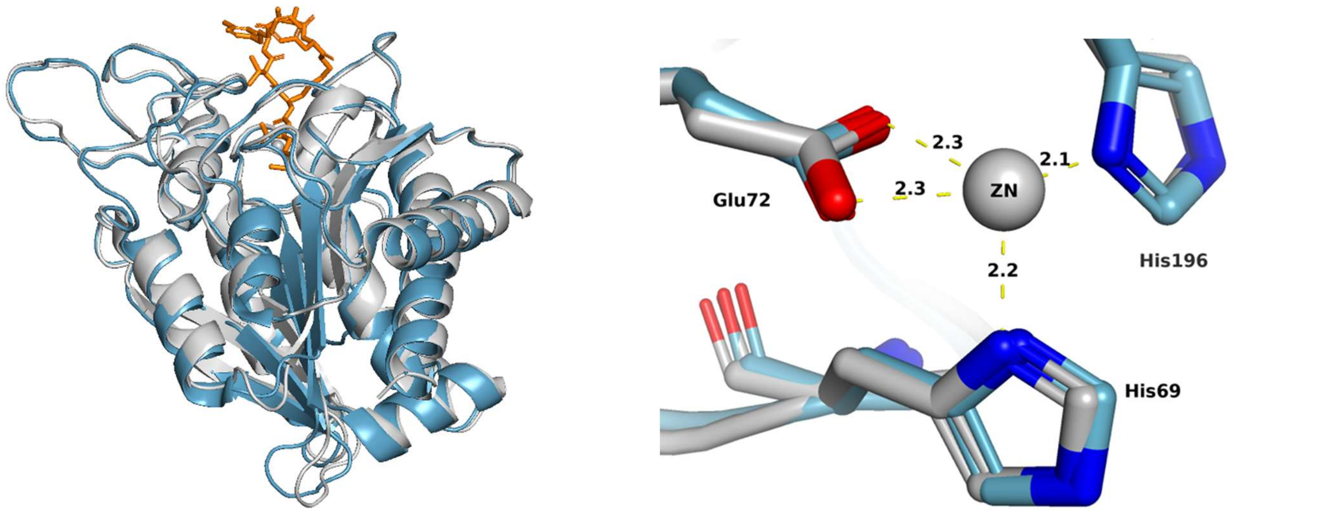

Figure S4: Overlay of CPA and CPB Active Sites Highlighting Conserved Active Site Residues. Structural comparison of CPA and CPB active sites. (**Left**) Overlay of CPA (PDB 3CPA, light blue)[2] and CPB bound to ABP C (PDB 5LRJ, gray)[1], showing the high degree of structural similarity between the two enzymes. ABP C (orange) extends out of the active site pocket. (**Right**) Zinc coordination environment in the CPB active site, highlighting the conserved residues His69, Glu72, and His196.

Table S1. Anabaenopeptin Type Natural Products and Source Organisms

Natural Product	Year	Source Organism	Source Type	Ref.
Anabaenopeptin	1995	*Anabaena* spp.	Cyanobacteria	3
Brunsvicamide	2006	*Tychonema* spp.	Cyanobacteria	4-6
Ferintoic Acid	1996	*Microcystis aeruginosa*	Cyanobacteria	7
Keramamide	1991	*Theonella* spp.	Sponge	8, 9
Konbamide	1991	*Theonella* spp.	Sponge	10
Lyngbyaureidamide	2012	*Lyngbya* spp.	Cyanobacteria	11
Mozamide	1997	*Theonella* spp.	Sponge	12
Namalide	2012	*Siliquariaspongia* / *Nostoc* spp.	Sponge / Cyanobacteria	13-15
Nodulapeptin	2011	*Nodularia spumigena*	Cyanobacteria	16-18
Nostamide	2017	*Nostoc* spp.	Cyanobacteria	15
Oscillamide	1998	*Oscillatoria* spp.	Cyanobacteria	19, 20
Paltolide	2010	*Theonella* swinhoei	Sponge	21
Psymbamide	2007	*Psammocinia* aff. *bulbosa*	Sponge	22
Schizopeptin	2002	*Schizothrix* spp.	Cyanobacteria	23

Explanatory Notes: Names of selected compounds (e.g., Paltolide, Keramamide, Konbamide) reflect geographic or biological origins. Not all Keramamides contain the ureido moiety and pentacyclic core, which appear restricted to Keramamides A and L.

Table S2. IC_{50} Values of Anabaenopeptins Against CPA (Converted to Molar Units)

Compound	MW (g/mol)	CPA IC_{50} (original)	µM	AA1	AA2	AA3	AA4	AA5	AA6	Ref
Anabaenopeptin 679	679.82	4.58 µg/mL / 6.74 µM	6.74	NH2	D-Lys	L-Val	L-Hty	L-MeAla	L-Phe	24
Anabaenopeptin 808*	807.99	< 25 µg/mL	< 30.94	Ile	Lys	Ile	Hty	MeAla	Phe	18
Anabaenopeptin 814	813.95	< 4 µg/mL	< 4.91	Phe	Lys	Val	Hty	MeGly	Phe	18
Anabaenopeptin 866	866.03	35 µg/mL	40.42	Ile	Lys	Val	Hph	MeHty	AcSer	18
Anabaenopeptin 868	868.10	45 µg/mL	51.84	Ile	Lys	Val	Hph	MeHty	Met	18
Anabaenopeptin 884	884.10	< 3 µg/mL	< 3.39	Ile	Lys	Val	Hph	MeHty	MetO	18
Anabaenopeptin 899	900.09	0.61 µM	0.61	Phe	Lys	Val	Hty	MeHty	Ile	25
Anabaenopeptin 900	900.16	< 28 µg/mL	< 31.13	Ile	Lys	Met	Hph	MeHty	Met	18
Anabaenopeptin 900	900.04	< 22 µg/mL	< 24.44	Phe	Lys	Val	Hph	MeHty	AcSer	18
Anabaenopeptin 908	909.10	> 10 µg/mL / > 11 µM	> 11	Arg	Lys	Val	Hty	MeHty	Ile	24
Anabaenopeptin 908	909.10	NA up to 20 µg/mL	**NA up to 22	Arg	Lys	Val	Hty	MeHty	Ile	26
Anabaenopeptin 915	916.09	0.12 µg/mL	0.13	Tyr	Lys	Val	Hty	MeHty	Ile	26
Anabaenopeptin 916*	916.04	< 4 µg/mL	< 4.37	Phe	Lys	Val	Hty	MeHty	AcSer	18
Anabaenopeptin 918	918.12	< 3 µg/mL	< 3.27	Phe	Lys	Val	Hph	MeHty	MetO	18
Anabaenopeptin 934	934.12	20 µg/mL	21.41	Phe	Lys	Val	Hty	MeHty	MetO	18
Anabaenopeptin A*	843.98	< 3 µg/mL	< 3.55	Tyr	Lys	Val	Hty	MeAla	Phe	18
Anabaenopeptin A*	843.98	< 3 µg/mL	< 3.55	Tyr	Lys	Val	Hty	MeAla	Phe	18
Anabaenopeptin SA6	835.02	4.5 µM	4.5	Ile	Lys	Leu*	Hph	MeAsn	Phe	27
Anabaenopeptin 806Ne	806.96	21.1 µM	21.1	Val	Lys	Leu*	Hph	Asn	Phe	27
Anabaenopeptin 820Ne	820.99	3.5 µM	3.5	Val	Lys	Leu*	Hph	MeAsn	Phe	27
Anabaenopeptin B	836.99	NA @ 50 µg/mL	NA @ 59.74	Arg	Lys	Val	Hty	MeAla	Phe	28
Anabaenopeptin B	836.99	3.9 µM	3.9	Arg	Lys	Val	Hty	MeAla	Phe	29
Anabaenopeptin B	836.99	> 20 µg/mL / > 24 µM	> 24	Arg	Lys	Val	Hty	MeAla	Phe	24
Anabaenopeptin C	808.98	> 100 µM	> 100	Lys	Lys	Val	Hty	MeAla	Phe	29
Anabaenopeptin D	827.98	< 3 µg/mL	< 3.62	Phe	Lys	Val	Hty	MeAla	Phe	18
Anabaenopeptin E	851.02	NA @ 50 µg/mL	NA @ 58.75	Arg	Lys	Val	MeHty	MeAla	Phe	28
Anabaenopeptin F	851.02	> 20 µg/mL / > 24 µM	> 24	Arg	Lys	Ile	Hty	MeAla	Phe	24
Anabaenopeptin F	851.02	NA @ 50 µg/mL	NA @ 58.75	Arg	Lys	Ile	Hty	MeAla	Phe	28
Anabaenopeptin F	851.02	1.1 µM	1.1	Arg	Lys	Ile	Hty	MeAla	Phe	29
Anabaenopeptin G	930.11	0.0018 µg/mL	0.001	Tyr	Lys	Ile	Hty	MeHty	Ile	28
Anabaenopeptin G	930.11	0.0070 µg/mL	0.007	Tyr	Lys	Ile	Hty	MeHty	Ile	30
Anabaenopeptin H	923.13	9.45 µg/mL / 10.2 µM	10.2	Arg	Lys	Ile	Hty	MeHty	Ile	24
Anabaenopeptin H	923.13	3.4 µg/mL	3.68	Arg	Lys	Ile	Hty	MeHty	Ile	28
Anabaenopeptin I	759.95	0.0052 µg/mL	0.00684	Ile	Lys	Val	Hty	MeAla	Leu	30
Anabaenopeptin J	793.96	0.0076 µg/mL	0.00957	Ile	Lys	Val	L-Hty	MeAla	Phe	30
Anabaenopeptin T	866.07	2.0 µg/mL	2.3	Ile	Lys	Val	L-Hty	MeHty	Ile	28
Anabaenopeptin T	866.07	0.022 µg/mL	0.0254	Ile	Lys	Val	Hty	MeHty	Ile	30
Brunsvicamide A (Synthetic)	845.06	0.0050 ± 0.0001 µM	0.0050 ± 0.0001	L-Ile	D-Lys	L-Val	L-Leu	MeTrp	L-Phe	6
Brunsvicamide A (Derivative 1)	845.06	0.0077 ± 0.0001 µM	0.0077 ± 0.0001	L-*allo*-Ile	D-Lys	L-Val	L-Leu	L-MeTrp	L-Phe	6
Brunsvicamide A (Derivative 2)	845.06	17.1 ± 0.5 µM	17.1 ± 0.5	D-*allo*-Ile	D-Lys	L-Val	L-Leu	L-MeTrp	L-Phe	6

Brunsvicamide A (Derivative 3)	845.06	27.1 ± 0.6 µM	27.1 ± 0.6	D-Ile	D-Lys	L-Val	L-Leu	L-MeTrp	L-Phe	6
Brunsvicamide A (Derivative 4)	845.06	5.4 ± 0.1 µM	5.4 ± 0.1	L-Ile	L-Lys	L-Val	L-Leu	L-MeTrp	L-Phe	6
Brunsvicamide A (Derivative 5)	845.06	> 50 µM	> 50	L-*allo*-Ile	L-Lys	L-Val	L-Leu	L-MeTrp	L-Phe	6
Brunsvicamide A (Derivative 6)	845.06	> 50 µM	> 50	D-*allo*-Ile	L-Lys	L-Val	L-Leu	L-MeTrp	L-Phe	6
Brunsvicamide A (Derivative 7)	845.06	> 50 µM	> 50	D-Ile	L-Lys	L-Val	L-Leu	L-MeTrp	L-Phe	6
Brunsvicamide A (Derivative 8)	802.97	0.102 ± 0.007 µM	0.102 ± 0.007	L-Ala	D-Lys	L-Val	L-Leu	L-MeTrp	L-Phe	6
Brunsvicamide A (Derivative 9)	802.97	5.7 ± 0.1 µM	5.7 ± 0.1	D-Ala	D-Lys	L-Val	L-Leu	L-MeTrp	L-Phe	6
Brunsvicamide A (Derivative 10)	768.96	0.0066 ± 0.0002 µM	0.0066 ± 0.0002	L-Ile	D-Lys	L-Val	L-Leu	L-MeTrp	L-Ala	6
Brunsvicamide A (Derivative 11)	798.98	0.0079 ± 0.0003 µM	0.0079 ± 0.0003	L-Ile	D-Lys	L-Val	L-Leu	L-MeTrp	L-Ser	6
Brunsvicamide A (Derivative 12)	729.92	0.0048 ± 0.0002 µM	0.0048 ± 0.0002	L-Ile	D-Lys	L-Val	L-Leu	L-MeAla	L-Phe	6
Brunsvicamide A (Derivative 13)	759.95	0.0289 ± 0.0005 µM	0.0289 ± 0.0005	L-Ile	D-Lys	L-Val	L-Leu	L-MeSer	L-Phe	6
Brunsvicamide A (Derivative 14)	802.97	0.0083 ± 0.0002 µM	0.0083 ± 0.0002	L-Ile	D-Lys	L-Val	L-Ala	L-MeTrp	L-Phe	6
Brunsvicamide A (Derivative 15)	833.00	0.0076 ± 0.0004 µM	0.0076 ± 0.0004	L-Ile	D-Lys	L-Val	L-Ser	L-MeTrp	L-Phe	6
Brunsvicamide A (Derivative 16)	817.00	0.0087 ± 0.0003 µM	0.0087 ± 0.0003	L-Ile	D-Lys	L-Ala	L-Leu	L-MeTrp	L-Phe	6
Brunsvicamide A (Derivative 17)	847.03	0.0225 ± 0.0015 µM	0.0225 ± 0.0015	L-Ile	D-Lys	L-Ser	L-Leu	L-MeTrp	L-Phe	6
Namalide (all L)	579.70	NA @ 30 µM	NA @ 30	L-Phe	L-Lys	L-Ile	L-Phe	-	-	13
Namalide (D-Lys)	579.70	0.25 ± 0.03 µM	0.25 ± 0.03	L-Phe	D-Lys	L-Ile	L-Phe	-	-	13
Namalide (L-allo-Ile)	579.70	*PR	*PR	L-Phe	D-Lys	L-*allo*-Ile	L-Phe	-	-	13
Namalide B	575.71	0.75 µM	0.75	Ile	Lys	Ile	Hty	-	-	14
Namalide C	561.68	2.0 µM	2	Ile	Lys	Val	Hty	-	-	14
Namalide dimer	~1158	NA @ 30 µM	NA @ 30	-	-	-	-	-	-	13
Namalide tetrapeptide	596.73	4.5 ± 0.9 µM	4.5 ± 0.9	Phe2	Lys	Ile	Phe1	-	-	13
Namalide Tricycle	388.51	NA @ 30 µM	NA @ 30	-	Lys	Ile	Phe1	-	-	13
Oscillamide Y	858.01	ND	ND	Tyr	Lys	Ile	Hty	MeAla	Phe	29
Oscillamide Y	858.01	15 µg/mL	17.48	Tyr	Lys	Ile	Hty	MeAla	Phe	18
Oscillamide Y*	858.01	15 µg/mL	17.48	Tyr	Lys	Ile	Hty	MeAla	Phe	18

Notes: Reported IC_{50} values in ng/mL were converted to µM using molecular weights from ChemDraw structures, assuming neutral forms. AA1–AA6 correspond to the general anabaenopeptin scaffold; stereochemistry is omitted for natural products and specified for synthetic variants. PR = poor reproducibility; NA@ = not active; ND = not determined. Values originally reported in µM are unchanged. *Denotes nodularin contamination.

Table S3. IC_{50} Values of Anabaenopeptins Against PP1 (Converted to Molar Units)

	Compound	MW (g/mol)	PP1 IC_{50} (original)	ng/mL	nM	µM	AA1	AA2	AA3	AA4	AA5	AA6	Ref
1	Anabaenopeptin 808*	807.99	55 ng/mL	55	68.07	0.068	Ile	Lys	Ile	Hty	MeAla	Phe	[18]
2	Anabaenopeptin 814	813.9530	435 ng/mL	435	534.43	0.534	Phe	Lys	Val	Hty	MeGly	Phe	[18]
3	Anabaenopeptin 866	866.0260	435 ng/mL	435	502.29	0.502	Ile	Lys	Val	Hph	MeHty	AcSer	[18]
4	Anabaenopeptin 868	868.1040	71 ng/mL	71	81.79	0.082	Ile	Lys	Val	Hph	MeHty	Met	[18]
5	Anabaenopeptin 870	870.0760	66 ng/mL	66	75.86	0.076	Phe	Lys	Val	Leu	MeHty	MetO	[18]
6	Anabaenopeptin 884	884.1030	53 ng/mL	53	59.95	0.060	Ile	Lys	Val	Hph	MeHty	MetO	[18]
7	Anabaenopeptin 900	900.1640	60 ng/mL	60	66.65	0.067	Ile	Lys	Met	Hph	MeHty	Met	[18]
8	Anabaenopeptin 900	900.0430	140 ng/mL	140	155.55	0.156	Phe	Lys	Val	Hph	MeHty	AcSer	[18]
9	Anabaenopeptin 916*	916.0420	50 ng/mL	50	54.58	0.055	Phe	Lys	Val	Hty	MeHty	AcSer	[18]
10	Anabaenopeptin 918	918.1200	100 ng/mL	100	108.92	0.109	Phe	Lys	Val	Hph	MeHty	MetO	[18]
11	Anabaenopeptin 934	934.1190	16 ng/mL	16	17.13	0.017	Phe	Lys	Val	Hty	MeHty	MetO	[18]
12	Anabaenopeptin A*	843.9790	88 ng/mL	88	104.27	0.104	Tyr	Lys	Val	Hty	MeAla	Phe	[18]
13	Anabaenopeptin A*	843.9790	86 ng/mL	86	101.90	0.102	Tyr	Lys	Val	Hty	MeAla	Phe	[18]
14	Anabaenopeptin A (lower)	843.9790	40% @ 0.009 µg/mL	9	10.1	0.011	Tyr	Lys	Val	Hty	MeAla	Phe	[31]
-	Anabaenopeptin A (upper)	843.9790	60% @ 4.5 µg/mL	-	-	5.33	Tyr	Lys	Val	Hty	MeAla	Phe	[31]
15	Anabaenopeptin B (lower)	836.9920	5% @ 0.009 µg/mL	9	10.1	0.011	Arg	Lys	Val	Hty	MeAla	Phe	[31]
-	Anabaenopeptin B (Upper)	836.9920	75% @ 4.5 µg/mL	-	-	5.38	Arg	Lys	Val	Hty	MeAla	Phe	[31]
16	Anabaenopeptin B	836.9920	9.5 ± 0.9 µM	-	-	9.5 ± 0.9	Arg	Lys	Val	Hty	MeAla	Phe	[32]
15	Anabaenopeptin D	827.9800	53 ng/mL	53	64.01	0.064	Phe	Lys	Val	Hty	MeAla	Phe	[18]
16	Anabaenopeptin F	851.0190	38.1% @ 100 µg/mL	-	-	-	Arg	Lys	Ile	Hty	MeAla	L-Phe	[33]
17	Anabaenopeptin F	851.0190	28.2 ± 3.4 µM	-	-	28.2 ± 3.4	Arg	Lys	Ile	Hty	MeAla	Phe	[32]
18	Oscillamide B	869.0520	43.3% @ 100 µg/mL	-	-	-	Arg	Lys	Met	Hty	MeAla	L-Phe	[33]
19	Oscillamide C	957.1430	97.3% @ 100 µg/mL; 0.90 µM	-	-	0.900	Arg	Lys	Ile	Hty	MeHty	L-Phe	[33]
20	Oscillamide Y	858.0060	11.2% @ 100 µg/mL	-	-	-	Tyr	Lys	Ile	Hty	MeAla	Phe	[33]
21	Oscillamide Y*	858.0060	62 ng/mL	62	72.26	0.072	Tyr	Lys	Ile	Hty	MeAla	Phe	[18]
22	Oscillamide Y	858.0060	62 ng/mL	62	72.26	0.072	Tyr	Lys	Ile	Hty	MeAla	Phe	[18]

Notes: Reported IC_{50} values in ng/mL were converted to µM using molecular weights calculated from ChemDraw structures, assuming neutral forms without counterions. Amino acid positions (AA1–AA6) correspond to the general anabaenopeptin scaffold; stereochemistry is omitted for clarity. Percent values indicate PP1 inhibition at the concentration reported in the original publication. When inhibition was reported as a range, upper and lower bounds are listed separately. Entries originally reported in µM are shown without further conversion. *Denotes nodularin contamination. Conversions correspond to Table 5 in the main text.

Table S4. Inhibition of PP2A by Anabaenopeptins and Related Peptides with Conversion to Molar Units

Anabaenopeptin	MW (g/mol)	% Inh. PP2A	Concentration Tested (µg/mL)	Calculated (µM)	AA1	AA2	AA3	AA4	AA5	AA6	Ref.
Anabaenopeptin F	851.0190	41.50%	100	117.5	Arg	Lys	Ile	Hty	MeAla	Phe	[33]
Oscillamide B	869.0520	62.80%	100	115.1	Arg	Lys	Met	Hty	MeAla	Phe	[33]
Oscillamide C	957.1430	98.40%	100	104.5	Arg	Lys	Ile	Hty	MeHty	Phe	[33]
Oscillamide Y	858.0060	9.60%	100	116.5	Tyr	Lys	Ile	Hty	MeAla	Phe	[33]

Notes: Reported inhibition values were based on treatment at 100 µg/mL unless otherwise noted. Test concentrations were converted to µM using molecular weights determined from ChemDraw structures, assuming neutral forms without counterions. Amino acid positions (AA1–AA6) correspond to the general anabaenopeptin scaffold; stereochemistry is omitted for natural products and specified for synthetic variants. Conversions correspond to Table 6 in the main text.

.to the chemical nature of the active center and the mechanisms of action. *Proc. Natl. Acad. Sci. U. S. A.* **1963**, 49 (1), 109-116. DOI: 10.1073/pnas.49.1.109.
(43) Reeck, G.

References

(1) Schreuder, H.; Liesum, A.; Lonze, P.; Stump, H.; Hoffmann, H.; Schiell, M.; Kurz, M.; Toti, L.; Bauer, A.; Kallus, C.; et al. Isolation, Co-Crystallization and Structure-Based Characterization of Anabaenopeptins as Highly Potent Inhibitors of Activated Thrombin Activatable Fibrinolysis Inhibitor (TAFIa). *Sci. Rep.* **2016**, 6 32958. DOI: 10.1038/srep32958.

(2) Christianson, D. W.; Lipscomb, W. N. X-ray crystallographic investigation of substrate binding to carboxypeptidase A at subzero temperature. *Proc. Natl. Acad. Sci. U. S. A.* **1986**, 83 (20), 7568-7572. DOI: 10.1073/pnas.83.20.7568.

(3) Harada, K.; Fujii, K.; Shimada, T.; Suzuki, M.; Sano, H.; Adachi, K.; Carmichael, W. W. Two cyclic peptides, anabaenopeptins, a third group of bioactive compounds from the cyanobacterium Anabaena flos-aquae NRC 525-17. *Tetrahedron Lett.* **1995**, 36 (9), 1511. DOI: 10.1016/0040-4039(95)00073-l.

(4) Muller, D.; Krick, A.; Kehraus, S.; Mehner, C.; Hart, M.; Kupper, F. C.; Saxena, K.; Prinz, H.; Schwalbe, H.; Janning, P.; et al. Brunsvicamides A-C: sponge-related cyanobacterial peptides with *Mycobacterium tuberculosis* protein tyrosine phosphatase inhibitory activity. *J. Med. Chem.* **2006**, 49 (16), 4871-4878. DOI: 10.1021/jm060327w.

(5) Walther, T.; Arndt, H. D.; Waldmann, H. Solid-support based total synthesis and stereochemical correction of brunsvicamide A. *Org Lett.* **2008**, 10 (15), 3199-3202. DOI: 10.1021/ol801064d.

(6) Walther, T.; Renner, S.; Waldmann, H.; Arndt, H. D. Synthesis and structure-activity correlation of a brunsvicamide-inspired cyclopeptide collection. *Chembiochem.* **2009**, 10 (7), 1153-1162. DOI: 10.1002/cbic.200900035.

(7) Williams, D. E.; Craig, M.; Holmes, C. F. B.; Andersen, R. J. Ferintoic acids A and B, new cyclic hexapeptides from the freshwater cyanobacterium *Microcystis aeruginosa*. *J. Nat. Prod.* **1996**, 59 (6), 570-575. DOI: DOI 10.1021/np960108l.

(8) Kobayashi, J.; Sato, M.; Ishibashi, M.; Shigemori, H.; Nakamura, T.; Ohizumi, Y. Keramamide A, a novel peptide from the Okinawan marine sponge *Theonella* sp. *J. Chem. Soc., Perkin Trans. 1* **1991**, (10), 2609.

(9) Uemoto, H.; Yahiro, Y.; Shigemori, H.; Tsuda, M.; Takao, T.; Shimonishi, Y.; Kobayashi, J. Keramamides K and L, new cyclic peptides containing unusual tryptophan residue from *Theonella* sponge. *Tetrahedron.* **1998**, 54 (24), 6719-6724. DOI: Doi 10.1016/S0040-4020(98)00358-5.

(10) Kobayashi, J. i.; Sato, M.; Murayama, T.; Ishibashi, M.; Wälchi, M. R.; Kanai, M.; Shoji, J.; Ohizumi, Y. Konbamide, a novel peptide with calmoduiin antagonistic activity from the Okinawan marine sponge *Theonella* sp. *J. Chem. Soc., Chem. Commun.* **1991**, (15), 1050-1052. DOI: 10.1039/c39910001050.

(11) Zi, J.; Lantvit, D. D.; Swanson, S. M.; Orjala, J. Lyngbyaureidamides A and B, two anabaenopeptins from the cultured freshwater cyanobacterium *Lyngbya* sp. (SAG 36.91). *Phytochemistry.* **2012**, 74 173-177. DOI: 10.1016/j.phytochem.2011.09.017.

(12) Schmidt, E. W.; Harper, M. K.; Faulkner, D. J. Mozamides A and B, cyclic peptides from a *theonellid sponge* from Mozambique. *J. Nat. Prod.* **1997**, 60 (8), 779-782. DOI: DOI 10.1021/np970195x.

(13) Cheruku, P.; Plaza, A.; Lauro, G.; Keffer, J.; Lloyd, J. R.; Bifulco, G.; Bewley, C. A. Discovery and synthesis of namalide reveals a new anabaenopeptin scaffold and peptidase inhibitor. *J. Med. Chem.* **2012**, 55 (2), 735-742. DOI: 10.1021/jm201238p.

(14) Sanz, M.; Salinas, R. K.; Pinto, E. Namalides B and C and Spumigins K-N from the Cultured Freshwater Cyanobacterium *Sphaerospermopsis torques-reginae*. *J. Nat. Prod.* **2017**, 80 (9), 2492-2501. DOI: 10.1021/acs.jnatprod.7b00370.

(15) Shishido, T. K.; Jokela, J.; Fewer, D. P.; Wahlsten, M.; Fiore, M. F.; Sivonen, K. Simultaneous Production of Anabaenopeptins and Namalides by the Cyanobacterium *Nostoc* sp. CENA543. *ACS Chem. Biol.* **2017**, 12 (11), 2746-2755. DOI: 10.1021/acschembio.7b00570.

(16) Fujii, K.; Sivonen, K.; Adachi, K.; Noguchi, K.; Sano, H.; Hirayama, K.; Suzuki, M.; Harada, K. Comparative study of toxic and non-toxic cyanobacterial products: Novel peptides from toxic *Nodularia spumigena* AV1. *Tetrahedron Lett.* **1997**, 38 (31), 5525-5528. DOI: Doi 10.1016/S0040-4039(97)01192-1.

(17) Mazur-Marzec, H.; Kaczkowska, M. J.; Blaszczyk, A.; Akcaalan, R.; Spoof, L.; Meriluoto, J. Diversity of peptides produced by *Nodularia spumigena* from various geographical regions. *Mar. Drugs.* **2012**, 11 (1), 1-19. DOI: 10.3390/md11010001.

(18) Spoof, L.; Blaszczyk, A.; Meriluoto, J.; Ceglowska, M.; Mazur-Marzec, H. Structures and Activity of New Anabaenopeptins Produced by Baltic Sea Cyanobacteria. *Mar. Drugs.* **2015**, 14 (1), 8. DOI: 10.3390/md14010008.

(19) Sano, T.; Kaya, K. Oscillapeptin G, a tyrosinase inhibitor from toxic Oscillatoria agardhii. *J. Nat. Prod.* **1996**, 59 (1), 90-92. DOI: 10.1021/np9600210.

(20) Marsh, I. R.; Bradley, M.; Teague, S. J. Solid-Phase Total Synthesis of Oscillamide Y and Analogs. *J. Org. Chem.* **1997**, 62 (18), 6199-6203. DOI: 10.1021/jo970671o.

(21) Plaza, A.; Keffer, J. L.; Lloyd, J. R.; Colin, P. L.; Bewley, C. A. Paltolides A--C, anabaenopeptin-type peptides from the palau sponge *Theonella swinhoei*. *J. Nat. Prod.* **2010**, 73 (3), 485-488. DOI: 10.1021/np900728x.

(22) Robinson, S. J.; Tenney, K.; Yee, D. F.; Martinez, L.; Media, J. E.; Valeriote, F. A.; van Soest, R. W.; Crews, P. Probing the bioactive constituents from chemotypes of the sponge *Psammocinia* aff. *bulbosa*. *J. Nat. Prod.* **2007**, 70 (6), 1002-1009. DOI: 10.1021/np070171i.

(23) Reshef, V.; Carmeli, S. Schizopeptin 791, a new anabeanopeptin-like cyclic peptide from the cyanobacterium *Schizothrix* sp. *J. Nat. Prod.* **2002**, 65 (8), 1187-1189. DOI: 10.1021/np020039c.

(24) Harms, H.; Kurita, K. L.; Pan, L.; Wahome, P. G.; He, H.; Kinghorn, A. D.; Carter, G. T.; Linington, R. G. Discovery of anabaenopeptin 679 from freshwater algal bloom material: Insights into the structure-activity relationship of anabaenopeptin protease inhibitors. *Bioorg. Med. Chem. Lett.* **2016**, 26 (20), 4960-4965. DOI: 10.1016/j.bmcl.2016.09.008.

(25) Bober, B.; Zmudzki, P.; Chrapusta-Srebrny, E. Occurrence of protease inhibitors in freshwater cyanobacterium *Woronichinia naegeliana* (Unger) Elenkin. *J. Phycol.* **2025**, 61 (1), 34-43. DOI: 10.1111/jpy.13527.

(26) Okumura, H. S.; Philmus, B.; Portmann, C.; Hemscheidt, T. K. Homotyrosine-containing cyanopeptolins 880 and 960 and anabaenopeptins 908 and 915 from *Planktothrix agardhii* CYA 126/8. *J. Nat. Prod.* **2009**, 72 (1), 172-176. DOI: 10.1021/np800557m.

(27) Konkel, R.; Grabski, M.; Ceglowska, M.; Wieczerzak, E.; Wegrzyn, G.; Mazur-Marzec, H. Anabaenopeptins from *Nostoc edaphicum* CCNP1411. *Int. J. Environ. Res. Public Health.* **2022**, 19 (19), DOI: 10.3390/ijerph191912346.

(28) Itou, Y.; Suzuki, S.; Ishida, K.; Murakami, M. Anabaenopeptins G and H, potent carboxypeptidase A inhibitors from the cyanobacterium *Oscillatoria agardhii* (NIES-595). *Bioorg. Med. Chem. Lett.* **1999**, 9 (9), 1243-1246. DOI: 10.1016/s0960-894x(99)00191-2.

(29) Halland, N.; Bronstrup, M.; Czech, J.; Czechtizky, W.; Evers, A.; Follmann, M.; Kohlmann, M.; Schiell, M.; Kurz, M.; Schreuder, H. A.; et al. Novel Small Molecule Inhibitors of Activated Thrombin Activatable Fibrinolysis Inhibitor (TAFIa) from Natural Product Anabaenopeptin. *J. Med. Chem.* **2015**, 58 (11), 4839-4844. DOI: 10.1021/jm501840b.

(30) Murakami, M.; Suzuki, S.; Itou, Y.; Kodani, S.; Ishida, K. New anabaenopeptins, potent carboxypeptidase-A inhibitors from the cyanobacterium *Aphanizomenon flos-aquae*. *J. Nat. Prod.* **2000**, 63 (9), 1280-1282. DOI: 10.1021/np000120k.

(31) Gkelis, S.; Lanaras, T.; Sivonen, K. The presence of microcystins and other cyanobacterial bioactive peptides in aquatic fauna collected from Greek freshwaters. *Aquat. Toxicol.* **2006**, 78 (1), 32-41. DOI: 10.1016/j.aquatox.2006.02.001.

(32) Bubik, A.; Frangez, R.; Zuzek, M. C.; Gutierrez-Aguirre, I.; Lah, T. T.; Sedmak, B. Cyanobacterial Cyclic Peptides Can Disrupt Cytoskeleton Organization in Human Astrocytes-A Contribution to the Understanding of the Systemic Toxicity of Cyanotoxins. *Toxins.* **2024**, 16 (9), DOI: 10.3390/toxins16090374.

(33) Sano, T.; Usui, T.; Ueda, K.; Osada, H.; Kaya, K. Isolation of new protein phosphatase inhibitors from two cyanobacteria species, *Planktothrix* spp. *J. Nat. Prod.* **2001**, 64 (8), 1052-1055. DOI: 10.1021/np0005356.
